# Assessing the effectiveness of ontology-grounded AI term extraction using OntoGPT for environmental evidence synthesis

**DOI:** 10.1186/s13750-026-00381-0

**Published:** 2026-02-08

**Authors:** Ryan Y. Hodgson, Steven A. Robinson, Amélie C. Boutin, Felix K. Chan, Joseph R. Bennett, Rachel T. Buxton, J. Harry Caufield, Dalal E. L. Hanna, Tim Alamenciak

**Affiliations:** 1 Department of Biology, Carleton University, 1125 Colonel By Drive, Ottawa, ON Canada; 2 Department of Geography and Environmental Studies, Carleton University, 1125 Colonel By Drive, Ottawa, ON Canada; 3 Environmental Genomics and Systems Biology, Lawrence Berkeley National Laboratory, 1 Cyclotron Road, Berkeley, CA 94720 USA; 4 University of Waterloo, Waterloo, Canada

**Keywords:** Evidence-based conservation, LLM, Structured data extraction, Ontology, Restoration ecology

## Abstract

Evidence syntheses are valuable sources of robust and transparent knowledge that can identify gaps in research and inform evidence-based decision making. However, the process of synthesis is time consuming and costly. We investigate a new AI-based method that uses a large-language model (LLM) grounded in ontologies (i.e. structured machine-interpretable glossaries of domain terminology) to extract information from a set of 80 articles on coastal wetland restoration outcomes. We evaluated this method by comparing human-extracted data with data extracted by OntoGPT — a Python package that combines an LLM with ontologies to extract structured information. We found that OntoGPT achieved 65% average agreement with human reviewers but varied based on information type requested for extraction. The highest agreement scores were found when extracting standardized information, and lower agreement scores were found for study-specific and interpretation-heavy information. Precision and recall — two common measurements of artificial intelligence performance — were 58% and 57%. Our results highlight the potential for LLMs to save some labour in the evidence synthesis process but highlight core challenges (e.g., complex information; subjective judgments) where further development is needed. While LLMs cannot replace human reviewers, they have the potential to assist in data extraction.

**Supplementary Information:**

The online version contains supplementary material available at 10.1186/s13750-026-00381-0.

## Introduction

 Evidence syntheses provide a valuable basis for environmental decision-making and research [1, 2]. Up-to-date and efficient syntheses help promote best management practices while keeping managers and practitioners informed on the best techniques and tools within a given field [3, 4]. However, evidence syntheses are labor-intensive, time consuming and costly, and face a continuously growing body of literature [4, 5]. Large language models (LLMs) are a type of artificial intelligence (AI) capable of summarizing and synthesizing text and can be a tool for automating evidence syntheses [6–8]. However, researchers have noted that LLMs can exhibit hallucinations, causing them to fabricate non-existent results and produce false answers [9]. Thus, there is keen interest in advancing methods that incorporate LLMs for data extraction and evidence synthesis, and a need for research that validates the effectiveness of these emerging tools.

OntoGPT is an open-source Python package with the potential to improve LLM-based data extraction through the integration of controlled vocabularies [10]. This process relies on existing publicly available knowledge bases called ontologies, which are created and collaboratively updated by domain experts [10, 11]. Ontologies contain information in an organized and structured format so that LLMs can interpret and link concepts together [11]. OntoGPT works by constraining LLM-based data extraction from source text to the concepts and terms defined within ontologies [10]. For example, the Environmental Ontology (ENVO) contains defined ecosystem types organized by key environmental features, specific ecological processes, and identifying characteristics [10, 11]. Ontologies are created by domain experts in collaboration with semantic engineers through community-driven, sometimes peer-reviewed development processes. Each ontology typically features continuous versioning, expert curation, and quality checks to ensure logical consistency and alignment with existing semantic frameworks. While the governance structures of ontologies vary, ENVO and other popular ontologies are community-run, with volunteer coordinators, and are collaboratively supported by the Open Biological and Biomedical Ontology (OBO) Foundry [12]. Ontologies can enhance the ability of LLMs to more accurately classify or identify concepts the model has not encountered previously, in contrast to other approaches that rely on lengthy fine-tuning steps where the LLM is trained on data in addition to its base dataset [10, 13]. However, there is a need for investigations that validate the performance of this novel approach for knowledge extraction [9].

To address this gap, we compared human-extracted information with information extracted by OntoGPT on a source literature sample of 80 scholarly articles about coastal wetland restoration. We outline and evaluate a method for incorporating automation in the extraction stage of an evidence synthesis using OntoGPT with human oversight.

### OntoGPT overview

OntoGPT is a Python package created to extract structured information from unstructured text [10]. The user specifies the information to be extracted by creating a template that defines the desired fields, relevant ontologies, and the output format of the extraction. The user supplies source text (e.g. a scholarly article) and OntoGPT generates structured prompts from the template that instruct an LLM to extract relevant terms from the source text. The extracted terms are then compared to ontology entries in a process known as “grounding.” This grounding step associates extracted terms (e.g. “wetland area”) with entries in an ontology (e.g. http://purl.obolibrary.org/obo/ENVO_00000043; “wetland area”). The OntoGPT recursively returns the results to the LLM to confirm they match the user-provided data fields. Users can specify which ontologies the script should consult or can create their own ontologies for the extraction. This approach enables LLMs to provide useful, accurate responses without the need to use task-specific training data. Previous text extraction approaches required large pre-training datasets to achieve similar results, but advances in LLM technology have made it possible for general models to respond effectively with a few prompts [14].

## Methods

We tested and validated this novel method of LLM-assisted data extraction on a source literature sample (*n* = 80) of scholarly articles on coastal wetland restoration outcomes. We analyzed the source literature using two methods, (1) human reviewers and (2) OntoGPT. We then compared both extractions with (1) an assessment of agreement by human reviewers, and (2) quantitative metrics of precision, recall, accuracy, and F1 score. These metrics enable a robust evaluation of both OntoGPT’s ability to collect correct information (precision), and its ability to capture all the requested information (recall), compared with the human reviewers’ extractions.

### Review dataset formation and manual dataset

We adapted the approach for systematic mapping [2] to collect a sample of source literature on outcomes of coastal wetland restoration for testing OntoGPT. Coastal wetland restoration was selected because it aligns with the expertise of the research leads and is an important issue in biodiversity conservation globally. Additionally, coastal wetlands were selected because they are a transitional zone with complex sources of degradation, which provide a wide variety of cases in which to test extraction [15]. Coastal wetland restoration also provides a diverse sample for OntoGPT, enhancing the applicability of our findings across environmental management.

We developed a search string based on the Society for Ecological Restoration’s definition of ecological restoration: “the process of assisting the recovery of an ecosystem that has been degraded, damaged, or destroyed” [16]. Our search was not limited to studies comparing restoration outcomes to a reference ecosystem standard, but instead encompassed a broad conceptualization of restoration including remediation, afforestation, rehabilitation, rewilding, and reclamation [17]. We searched three platforms (Web of Science, Scopus and Google Scholar) and modified our queries to accommodate the advanced search options of each platform (see Additional file 1 for search strings). We incorporated terms such as “success,” “failure,” “monitoring,” “biomonitoring,” “recovery,” and “status report” to focus on studies documenting restoration outcomes and limited the scope to journal articles published between 2009 and 2024. Each platform was searched individually, and all search results were uploaded and deduplicated in Rayyan [18] (an online tool for organizing systematic reviews), yielding an initial screening set of 1,829 items. Further review yielded 496 articles that met al.l inclusion criteria (see Additional file 2 for inclusion criteria). We selected 80 articles at random to test the extraction methods (see Additional file 3 for a bibliography of the source literature sample). For consistency, we use the term “source literature” to refer to the full-text content of each included article. The number of articles was set at 80 to match the available capacity for extraction, validation and comparison. We stratified the random sample by date to ensure representation of the range of dates in the larger source literature dataset. This sample yields a margin of error for representation of +/- 10% at a 95% confidence interval, using the formula $\:MOE=\frac{z*\sqrt{p*\left(1-p\right)}}{\sqrt{\left(N-1\right)*n/(N-n)}}$, where z is a constant of 1.96 for a confidence level of 95%, p is the proportion of the sample (set to 50% for a conservative estimate), N is the total population size (496) and n is the sample size (80). See Additional file 4 for more details on the literature search and screening process.

We developed 11 attributes as the targets for data extraction, including study site, geographic coordinates of restoration sites, ecosystem types, restoration actions, restoration start date, restoration end date, sampling and monitoring methods, monitoring start date, monitoring end dates, focal species, and response variables (see Table 1 for attribute definitions).

Manual data extraction was performed individually by AB, FC, RH and SR. Prior to the full extraction, manual reviewers extracted terms from the same 10 source literature articles and discussed their extractions. During the manual extraction, we retained the authors’ terminology for all attributes (e.g., ecosystem type, restoration action), but we did not extract full verbatim sentences. Instead, reviewers synthesized each attribute using the specific terms used in the source literature [19]. This strategy and the attributes selected allowed us to document the diversity of outcomes of restoration (response variables), strategies employed to measure these outcomes (monitoring and sampling methods), and the form of restoration (restoration actions) that they were linked to.

### AI-driven data extraction (OntoGPT)

**Fig. 1:**
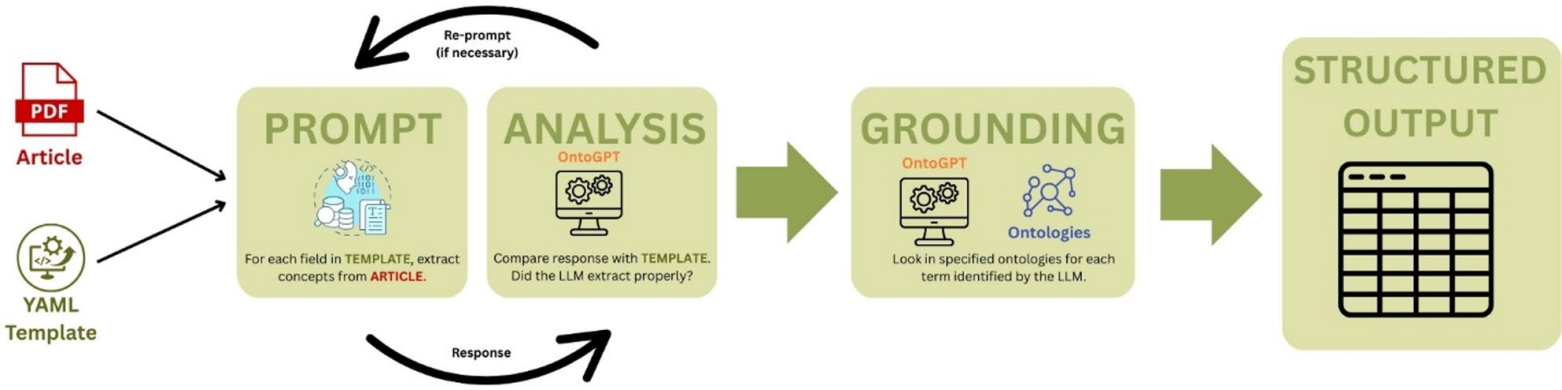
OntoGPT grounding process. OntoGPT takes text, a template and ontologies as input. It prepares prompts for an LLM (in this case, ChatGPT). The prompts request that the LLM extracts information from the PDF that respond to the fields outlined in the template. OntoGPT then evaluates the response from the LLM to ensure that the responses are appropriate to the template (e.g. the data type matches the field’s data type). OntoGPT then grounds the response of the LLM by searching the ontologies indicated in the template for all terms. Finally, it produces a structured output in the desired format (e.g. CSV)

We selected OntoGPT as the tool for data extraction because it is open source, freely available and has been tested in the biomedical field, three factors which align with the Responsible AI in Evidence Synthesis (RAISE) criteria for tool selection [20]. While there are other tools to facilitate data extraction, to our knowledge OntoGPT is the most developed package that integrates ontologies with an LLM to annotate text. OntoGPT’s default model is OpenAI’s GPT-4o (used for this extraction), though it supports a variety of other LLMs. OntoGPT is run through a Python command-line interface and relies on schema files written in YAML. Users provide a text input (either a PDF, plaintext or a URL) and a schema template that contains a list of ontologies to use for grounding. In this case, PDFs were provided to OntoGPT, whereupon it parses them to plain text, constructs prompts based on the YAML template and queries the LLM with the text and prompts. The extracted terms are matched to ontologies using string similarity, and the LLM is re-prompted to correct any mismatches or missing fields. The output is given in CSV format (Fig. 1).

We selected three well-developed general environment ontologies for the extraction: the Environmental Thesaurus Ontology, Environmental Ontology, and the Semantic Web for Earth and Environment Technology Ontology (see Table 2 for citations). We selected more specific ontologies for certain attributes: Gazeteer, a geographic ontology, was used for study site, and three taxonomic ontologies were used for species: the Plant Diversity Ontology, NCBI Taxon Ontology and the Vertebrate Taxonomy Ontology (Table 2). Relatively few suitable ontologies were available for the chosen attributes, so a formal selection process was not required. However, when multiple options existed we included them to maximize coverage and to enable comparison between ontologies.

Following the Structured Prompt Interrogation and Recursive Extraction of Semantics (SPIRES) methodology [10], we developed data extraction instructions for OntoGPT in the form of a YAML file (herein: extraction template) that described these extraction fields and drew on a set of eight environment, biology and ecology related ontologies (see Additional file 5). Our extraction template included a descriptive prompt and corresponding ontologies in which to ground term extraction (Table 1). OntoGPT used the template to extract data from the PDF files of the source literature sample using OpenAI’s GPT-4o LLM.

The structured output includes the ontology entry used for each extracted value and its associated attribute. In some cases, OntoGPT extracts values that are not contained in an ontology but still match the prompt description. In these situations, the software package will label values as being auto-generated [10]. Additionally, within our extraction template we included statistical methods as an attribute for extraction, but it was not included in our comparison as no manual extraction was conducted for this attribute. Manual reviewers found the category to be too broad, since even basic procedures like calculating the mean of a set of numbers could conceivably be “statistical methods.”

**Table 1:** Descriptions of attributes used in manual and OntoGPT data extraction comparisons, along with their associated ontologies

Attribute	Description	Ontology
Study site	The geographical location where restoration and the study took place.	GAZ ENVTHES
Latitude/longitude	Geographic coordinate locations of sampling or monitoring.	-
Ecosystem type	The ecosystem the restoration and monitoring or sampling occurred in.	ENVO SWEET ENVTHES
Restoration actions	The specific restoration actions being taken as part of the study.	ENVO ENVTHES
Sampling and monitoring methods	Methods implemented to monitor restoration.	ENVO ENVTHES SWEET
Focal species	Species restored or monitored that directly relate to outcome of restoration.	NCBITaxon VTO PDO_CAS
Response variables	Measurable variable observed during monitoring, that reflects the outcome of restoration action.	ENVO SWEET ENVTHES
Restoration start date	Dates when restoration started.	-
Restoration end date	Dates when restoration ended.	-
Monitoring start date	Dates when monitoring started.	-
Monitoring end date	Dates when monitoring started.	-

The OBI and STATO ontologies were also integrated into the script to extract statistical methods; however, this attribute was not addressed in manual extraction and is not included in the extraction comparisons

**Table 2:** Ontology prefixes, full names, and sources used in script development

Ontology prefixes	Ontology name	Ontology repository	Web address
SWEET	Semantic Web for Earth and Environment Technology Ontology	BioPortal	[21]
ENVO	Environmental Ontology	OBO	[22]
ENVTHES	Environmental Thesaurus Ontology	BioPortal	[23]
GAZ	Gazetteer	BioPortal	
OBI	Ontology for Biomedical Investigation	OBO	[24]
STATO	Statistics Ontology	OBO	[25]
NCBITaxon	National Centre for Biotechnology Information Organismal Taxonomy	OBO	[26]
VTO	Vertebrate Taxonomy Ontology	OBO	[27]
PDO_CAS	Plant Diversity Ontology	BioPortal	[28]

*BioPortal* National Center for Biomedical Ontology Bioportal, *OBO* Open Biological and Biomedical Ontology

### Comparison of manual and OntoGPT data extraction

For each source literature, manually extracted data was compared with OntoGPT-extracted outputs by manually scoring agreement for each attribute (Table 3). Agreement was classified as either “agreement,” “partial agreement,” or “disagreement,” and assigned a score of 2, 1 or 0, respectively. A score of two indicated that manual and automated extractions were in full agreement, with some leeway for minimal differences in semantic expression if the terms could still be evaluated as meaning the same thing (e.g., saline marsh vs. salt marsh; or tidal reinstatement vs. tidal flow restoration). A score of 1 indicated partial agreement, where at least one piece of information agreed between both methods of extraction. A score of 0 indicated disagreement, and all extracted values did not agree. The numerical values corresponding to the assigned level of agreement were summed to calculate the overall agreement for each attribute, expressed as the percentage of the maximum possible score of 160 (score of “2” for each attribute across the 80-article sample).

To compare the volume of data generated by manual and OntoGPT extractions, we tallied the number of entries associated with each attribute for both methods. An entry was counted as one term regardless of the number of words it contained, and “none reported” was recorded in cases where there was no entry for an attribute. Thus, for both manual and OntoGPT extractions, each source literature had at least 11 entries (one for each attribute), and each attribute had at least 80 entries (one for each article in the sample).

Furthermore, based on OntoGPT’s output for non-date attributes, we quantified the number of extractions associated with each ontology to determine which were most frequently used.

### Performance metrics: precision, recall, accuracy, and F1 score

We calculated common metrics of AI evaluation: precision (positive predictive value), recall (sensitivity, true positive rate), accuracy (percent agreement), and F1 score [29]. All metrics were calculated for OntoGPT extractions relative to human extraction baseline using term matching. Extraction outcomes were classified as true positives (TP is the number of occasions where OntoGPT returned an output that matched the manually extracted value), false positives (FP is the number of occasions where OntoGPT returned an output that did not match any manually extracted value), true negatives (TN is the number of occasions where OntoGPT correctly returned no output because the required information was not present in the source literature), and false negatives (FN is the number of occasions where the manual extraction identified information that OntoGPT failed to extract). We then tallied TP, FP, TN, and FN for each attribute to generate confusion matrices for metric calculation. Precision $\:\left(\frac{TP}{TP+FP}\right)$ is the proportion of positive OntoGPT extractions that were correct [30]. Recall $\:\left(\frac{TP}{TP+FN}\right)$ is the proportion of all relevant information that OntoGPT successfully identified [30]. Accuracy $\:\left(\frac{TP+TN}{TP+TN+FP+FN}\right)$ is the proportion of all OntoGPT extractions (positive and negative) that were correct. F1 score is a combination of precision and recall and is calculated as the harmonic mean of these two metrics $\:\left(2*\frac{precision\:*\:recall}{precision\:+\:recall}\right)$ [29]. F1 score captures the importance of extracting all relevant information (perfect recall) while minimizing irrelevant information (perfect precision). Each metric ranges from 0 to 1, with 1 indicating perfect extraction performance and 0 indicating the worst model performance.

Geographic coordinates and date-based attributes were assessed with manual oversight of term matching to accommodate the variety of formats encountered and the prevalence of special character text in both manual and OntoGPT extractions. Geographic coordinates were converted to a single format and parsed to decimal degrees with the *parzer* R package [31] and matched for TP within a margin of ± 0.01 degrees. Date-based attributes were matched as TPs when there was agreement on the year, since many studies only report the year when describing restoration or monitoring efforts. Extractions for term-based attributes (study sites, ecosystem types, restoration actions, sampling and monitoring methods, focal species, and response variables) were matched at the word level by splitting extractions into single-word tokens with *tidytext* package [32]. After tokenization, common words (e.g., the, of, and) were removed with the *stopwords* package [33]. To improve matching, tokens for ecosystem type, restoration actions, monitoring methods, and response variable attributes were stemmed to their root with *SnowballC* package [34], while study sites and focal species were not stemmed to maintain the distinctiveness of place names and to accommodate Latin nomenclature (they were, however, tokenized to split the extractions into single words). To reduce noise in the matching process, we filtered out one- and two-character terms and prioritized terms with the same three-character prefix. Fuzzy matching of tokens and stems was completed with *fuzzyjoin* [35] and *stringdist* [36] packages, and TP matches were determined using a Jaro-Winkler string distance threshold of 0.15 (85% similarity) for matches with identical prefixes, and 0.05 (95% similarity) for matches with different prefixes. To account for duplicate tokens or stems, we adopted a multiset matching approach where the maximum number of TP matches for a given paper and attribute combination was limited to the frequency of the term in the manual extraction – OntoGPT duplicates above this cap were treated as FP. After initial tests of the fuzzy matching workflow, a small synonym dictionary was introduced to address three obvious cases where equivalent terms or compound terms were not being matched: “salt / saline”, “seagrass / sea grass”, and “saltmarsh / salt marsh”.

To determine OntoGPT’s overall extraction performance, we summarized metrics into macro (unweighted) and micro (weighted) averages. A macro-average (arithmetic mean) assigns equal weight to each attribute, whereas a micro-average is weighted by attribute support – the number of attribute occurrences identified in manual extraction (TP + FN) [37, 38]. Attributes with larger support will have a greater influence on the micro-average. Though each metric provides insight into OntoGPT’s extraction performance, we chose to emphasize precision as it is not influenced by FN outcomes and most directly indicates OntoGPT’s ability to extract correct information.

Metric analysis was completed with *R* version 4.4.3 [39] in the *RStudio* IDE [40]. Data files and R scripts for data cleaning, fuzzy term matching, and metric calculation are provided in Additional file 6.

## Results

### Agreement between manual and OntoGPT data extraction

**Fig. 2:**
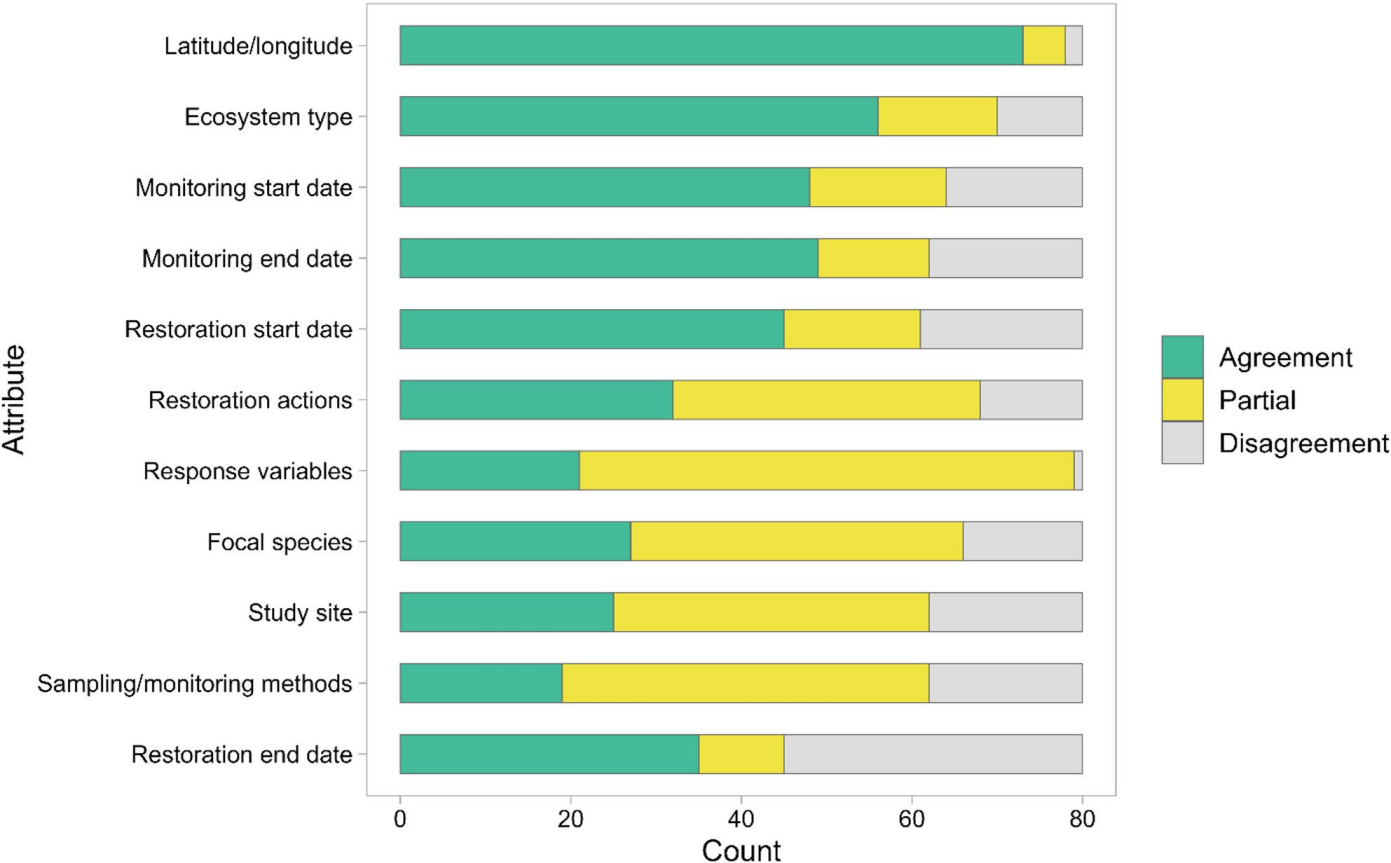
Number full agreement, partial agreement, or disagreement between manual and OntoGPT extractions for 11 attributes from a sample (*n* = 80) of coastal wetland restoration source literature based on reviewer comparison. Attributes are presented by descending agreement score

Extraction agreement ranged from a maximum of 94% (latitude/longitude) to a minimum of 50% (restoration end date). The average agreement score across all attributes was 65%. In general, the highest agreement scores were noted for less complex attributes such as latitude/longitude (94% agreement) and ecosystem type (79%), while lower agreement was attained for more complex fields such as sampling/monitoring methods (51%), restoration action (63%), and response variables (63%). Similarly, complex attributes had a larger percentage of partial agreement between manual and OntoGPT extractions (e.g., response variables and sampling and monitoring methods, 73% and 54% partial agreement, respectively). Response variable was the attribute with the fewest disagreement ratings (1%), yet also the second fewest full agreement ratings (26%). Date-based attributes showed a range of agreements, with monitoring start date (70%) and monitoring end date (69%) scoring higher than restoration start date (66%) and restoration end date (50%). See Table 3 for an example comparison and Table 4; Fig. 2 for full agreement results.

To determine whether and to what degree OntoGPT hallucinated (i.e. returned information that did not appear anywhere in the source literature), we examined all manual extractions for which the reviewer had extracted “none reported” (*n* = 102). Of those, OntoGPT also extracted “none reported” for 68 results. We reviewed all mismatches where the manual reviewer extracted nothing and OntoGPT found something (*n* = 35). Of those, 28 records were related to restoration and monitoring dates, 4 were for the focal species, 1 was for restoration actions and 2 for the study site coordinates. With respect to the dates, many articles in the source literature do not explicitly distinguish between dates for monitoring and dates for restoration, which could have caused these errors. All dates extracted did appear in the source literature but may not have been explicitly associated with restoration or monitoring. With respect to focal species (*n* = 4), all species picked up by OntoGPT were mentioned in the source literature, though may not have been the focus of restoration efforts. One species was an incorrect taxon, but the genus was present in the source literature. The disagreement for the restoration action category was from an article about mangrove restoration, where the authors did not explicitly state that mangroves were planted, so the manual reviewer did not extract anything, but OntoGPT inferred correctly that the restoration action was “plant mangroves.” In the case of study coordinates, OntoGPT extracted a string of numbers that looked coordinate-like in one case, and in another case, it correctly extracted coordinates where the manual reviewer had made an error. All disagreements were still grounded in the text, and OntoGPT did not appear to hallucinate answers (i.e., produce outputs not in the source text) in any of the cases where no information was present.

**Fig. 3:**
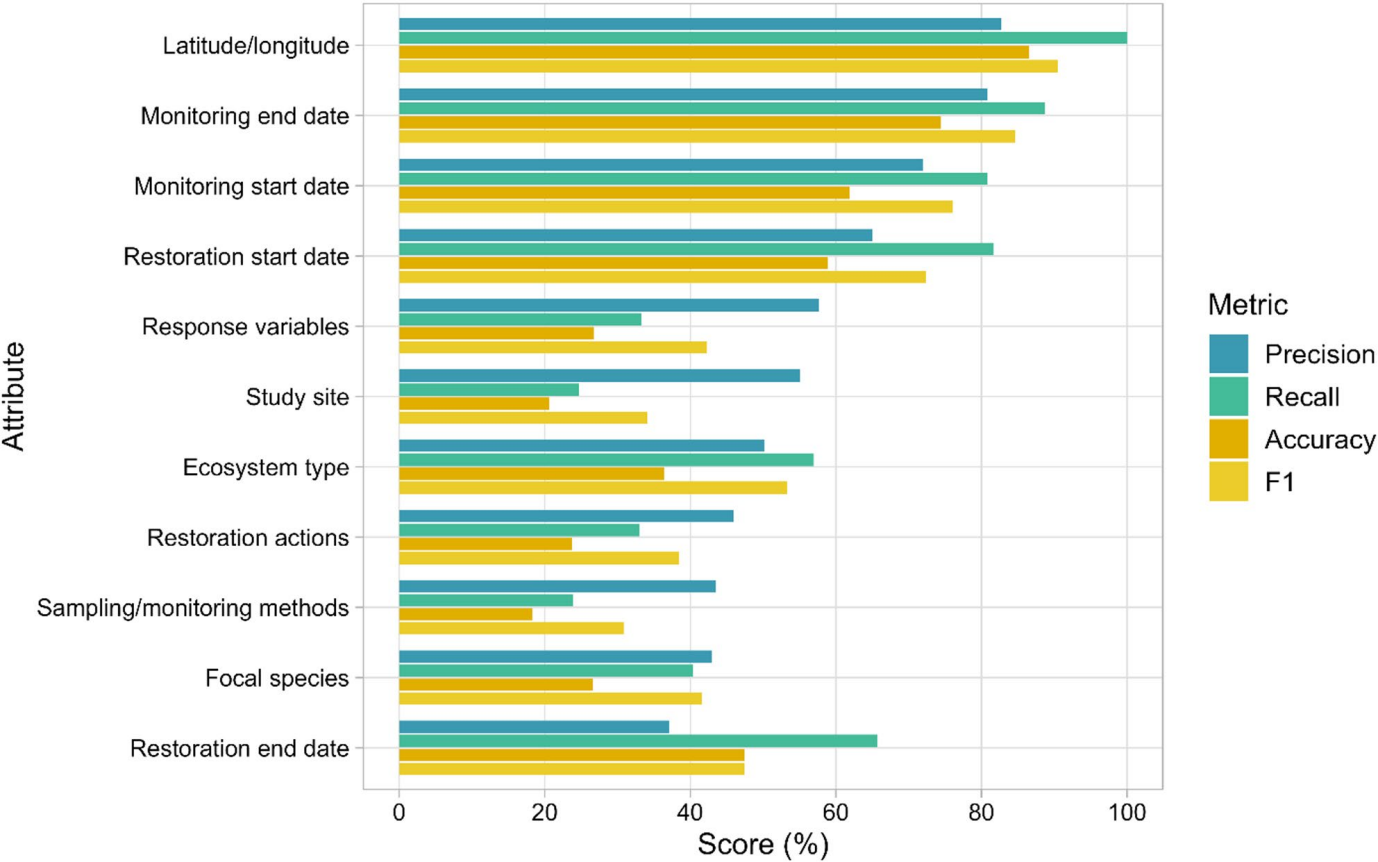
OntoGPT data extraction performance metrics (precision, recall, accuracy, F1 score), presented by descending precision

We limited the scope of manual extractions to text as we did not expect OntoGPT to extract information from tables and figures. However, during agreement assessments, we found that OntoGPT had pulled accurate geographic coordinates (22 total) from tables in three source literature items. We added these coordinates as true positives in our manual extractions but did not screen tables from other source literature. The remainder of the attributes were found in the source literature and compared effectively (Fig.3).

**Table 3:** Example of agreement assessment between human reviewer and OntoGPT data extractions for 5 selected attributes in thiet et al. (2014)

Attribute	Manual extraction	OntoGPT extraction	Agreement score
Ecosystem type	salt marsh lagoon	salt marsh	2
Restoration actions	restored connectivity partial tidal restoration	Restoration flow	2
Focal species	molluscs Mya arenaria Mercenaria mercenaria Spisula solidissma	Mya Mercenaria Macoma balthica	1
Sampling and monitoring methods	molluscan surveys benthic core samples water temperature (C) salinity (ppt)	Mollusks measurement sediment aquatic vegetation	1
Response variables	molluscan species richness molluscan density (m^2^) submerged aquatic vegetation density (SAV)	species richness density temperature of water salinity sediment	1

An agreement score was assigned for each attribute: 2 = agreement, 1 = partial agreement, and 0 = disagreement. See Additional file 7 for further examples

**Table 4:** Qualitative agreement between manual and OntoGPT extracted data by restoration attribute for *n* = 80 coastal wetland restoration source literature sample

Attribute	Frequency			Agreement (%)
	Agreement	Partial	Disagreement	
Latitude/longitude	73	5	2	94.4
Ecosystem type	56	14	10	78.8
Monitoring start date	48	16	16	70.0
Monitoring end date	49	13	18	69.4
Restoration start date	45	16	19	66.3
Response variables	21	58	1	62.5
Restoration actions	32	36	12	62.5
Focal species	27	39	14	58.1
Study site	25	37	18	54.4
Sampling/monitoring methods	19	43	18	50.6
Restoration end date	35	10	35	50.0
All attributes	430	287	163	65.2

Agreement is the sum of weighted agreement (full agreement = 2 points, partial agreement = 1 point), expressed as a percentage of the total possible score (160) for each attribute

Attributes varied in complexity, based on the number of possible entries and the degree of interpretation involved in extraction. More complex attributes had a lack of standardization of extracted terms (i.e., high variation in phrases and wordings used to describe the same or similar terms), required interpretation (extraction often required understanding multiple terms or concepts), and were context dependent (included study specific protocols, equipment, timing and/or conditions). For example, sampling and monitoring methods were relatively complex with methods often being highly study-specific and detailed, compared to latitude/longitude that had standardized formats for reporting across studies (Fig. 3).

### Precision, recall, accuracy, and F1 score

**Fig. 4:**
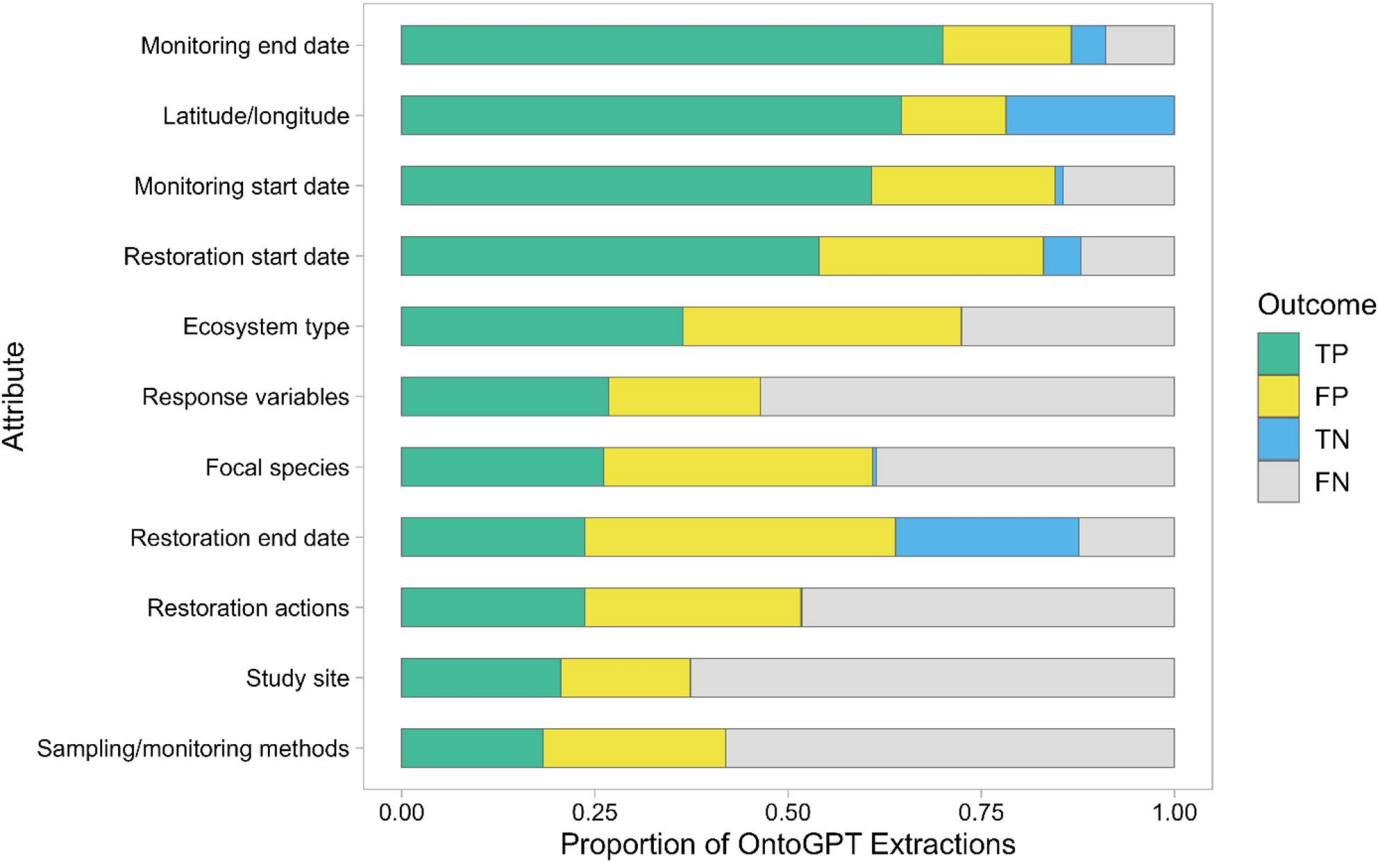
Proportion of OntoGPT data extraction outcomes matched as true positives (TP), false positives (FP), true negatives (TN), and false negatives (FN) relative to human extractions. Attributes are presented by descending TP proportion

From our analysis of OntoGPT extraction performance (Table 5), we calculated overall macro-averages of precision: 58%, recall: 57%, accuracy: 44%, and F1 score: 56%. Weighted micro-averages were lower for all metrics (precision: 52%, recall: 36%, accuracy: 28%, F1 score: 43%). For individual attributes, metric ranges were: precision 37%-83%, recall 24%-100%, accuracy 18%-87%, and F1 score 31%-91% (Fig. 4). Apart from restoration end date (which had the lowest precision of all attributes), value-based attributes had higher precision than term-based attributes, with latitude/longitude (83%), monitoring end date (81%), monitoring start date (72%), and restoration start date (65%) scoring highest on this metric. In general, attributes with a higher number of manual extractions scored lower on recall and accuracy, reflecting the higher proportion of FN outcomes observed for these attributes (Fig. 4).

TP matches for all attributes are provided in Additional file 8, unmatched terms from fuzzy matching (FP and FN) are provided in Additional file 9, and evaluation outcomes for dates (“date_tally.csv”) and latitude/longitude (“coord_tally.csv”) comparisons are provided in Additional file 6.

**Table 5:** OntoGPT data extraction outcomes and performance metrics relative to manual extraction

Attribute	Number of manually extracted items	Extraction Outcome				Metric (%)			
		TP	FP	FN	TN	Precision	Recall	Accuracy	F1
Latitude/longitude	86	86	18	0	29	82.7	100.0	86.5	90.5
Monitoring end date	71	63	15	8	4	80.8	88.7	74.4	84.6
Monitoring start date	73	59	23	14	1	72.0	80.8	61.9	76.1
Restoration start date	82	67	36	15	6	65.0	81.7	58.9	72.4
Response variables	1242	414	304	828	0	57.7	33.3	26.8	42.2
Study site	659	163	133	496	0	55.1	24.7	20.6	34.1
Ecosystem type	218	124	123	94	0	50.2	56.9	36.4	53.3
Restoration actions	528	174	205	354	1	45.9	33.0	23.8	38.4
Sampling/monitoring methods	873	209	271	664	0	43.5	23.9	18.3	30.9
Focal species	557	225	299	332	4	42.9	40.4	26.6	41.6
Restoration end date	35	23	39	12	23	37.1	65.7	47.4	47.4
Total	4424	1607	1466	2817	68				
Macro-average						57.5	57.2	43.8	55.6
Micro-average						52.3	36.3	28.1	42.9

Attributes are presented by descending precision

Each OntoGPT extraction was classified as a true positive (TP), false positive (FP), true negative (TN), or false negative (FN). Precision $\:=\frac{TP}{TP+FP}$; recall $\:=\frac{TP}{TP+FN}$; accuracy $\:=\frac{TP+TN}{TP+FN+FP+FN}$; F1 score $\:=2*\frac{precision\:*\:recall}{precision\:+\:recall}$. Macro-average is the unweighted arithmetic mean, and micro-average is weighted by the number of items from manual extractions (TP+FP)

### Volume of extracted data

**Fig. 5:**
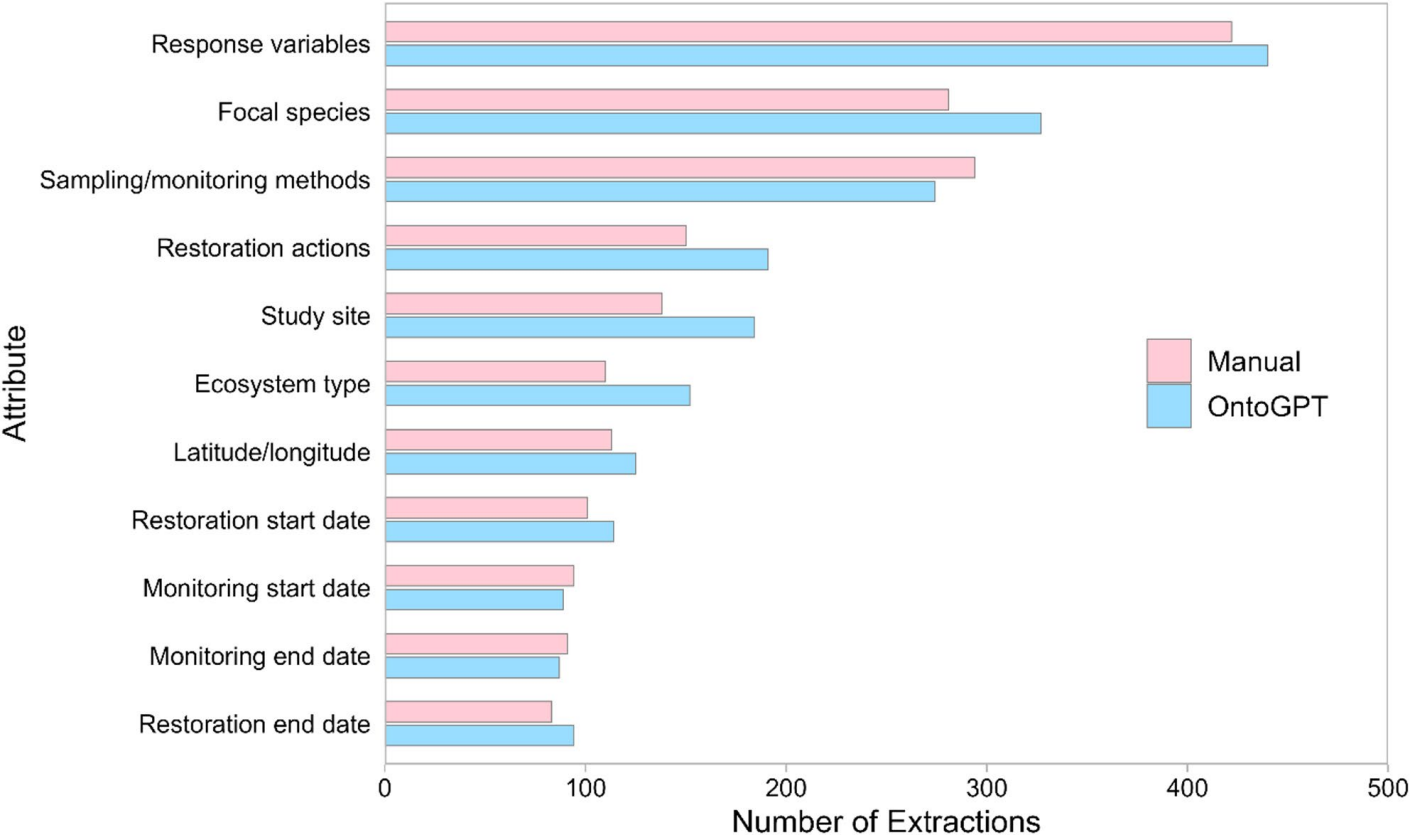
Total number of attribute extractions from source literature sample by manual and OntoGPT methods

OntoGPT extracted 2.5 more entries per source literature item than manual reviewers and more entries for each attribute except sampling and monitoring methods, monitoring start date, and monitoring end date (Fig. 5). Response variables (22%), focal species (15%), and sampling/monitoring methods (14%) combined for more than half of the 3,954 total entries obtained from both methods (Table 6).

**Table 6:** Number of attribute extractions by manual and OntoGPT methods for *n* = 80 coastal wetland restoration sample

Attribute	Extractions		Extractions per Source Item		
	Manual	OntoGPT	Manual	OntoGPT	Difference
Study site	138	184	1.7	2.3	+ 0.6
Latitude/longitude	113	125	1.4	1.6	+ 0.2
Ecosystem type	110	152	1.4	1.9	+ 0.5
Restoration actions	150	191	1.9	2.4	+ 0.5
Restoration start date	101	114	1.3	1.4	+ 0.1
Restoration end date	83	94	1	1.2	+ 0.2
Sampling/monitoring methods	294	274	3.7	3.4	− 0.3
Monitoring start date	94	89	1.2	1.1	− 0.1
Monitoring end date	91	87	1.1	1.1	–
Focal species	281	327	3.5	4.1	+ 0.6
Response variables	422	440	5.3	5.5	+ 0.2
All attributes	1877	2077	23.3	26.0	+ 2.5

### OntoGPT ontology usage

**Fig. 6:**
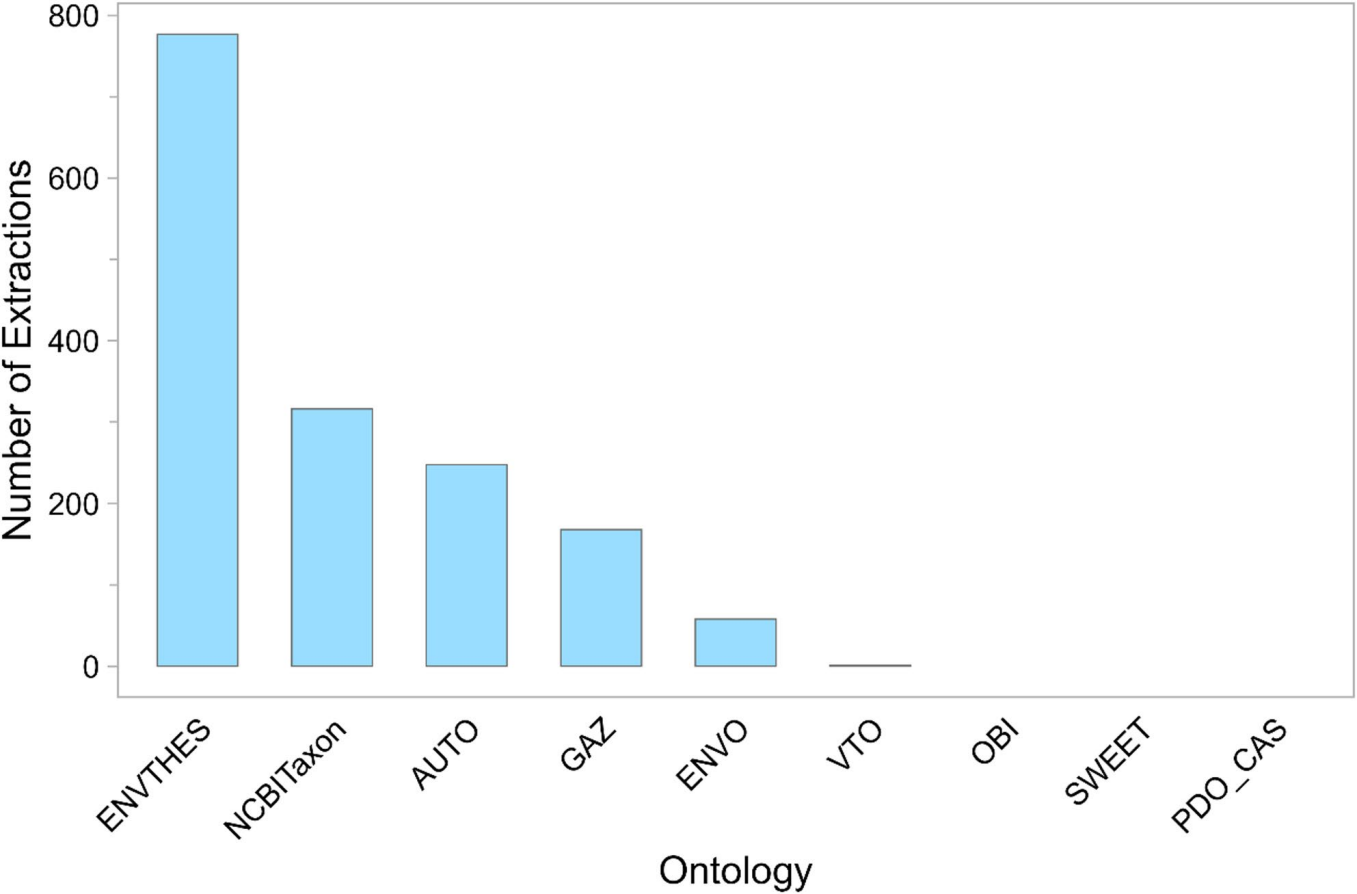
Number of extractions by OntoGPT for non-date attributes based on source ontology. “AUTO” represents values extracted from source literature that were not grounded in an ontology. OBI, SWEET, and PDO_CAS did not yield any extractions. See Table 2 for ontology abbreviations

ENVTHES was by far the most utilized ontology, comprising 50% of all OntoGPT extractions for attributes that were associated with an ontology (Fig. 6). NCBITaxon was used for 20% of extractions, followed by GAZ (11%) and ENVO (4%). Only a single extraction was obtained from VTO, while SWEET, OBI, and PDO_CAS yielded no extractions at all. Self-generated terms (AUTO) represented 16% of all OntoGPT attribute extractions. The goals and composition of the ontologies may have played a substantial role in determining which were more used than others.

### OntoGPT stability

The initial term extraction was conducted December 2024 and comparison with manual screening proceeded immediately after. To assess the inherent variability of OntoGPT, we ran multiple replicates of the extraction in March 2025. Results were consistent within the replicant group (average agreement: 99.4%). However, when compared with the group extracted three months prior, there was an average agreement of 72.4%. Using fuzzy matching from *fedmatch* R package [41], we found a slightly higher level of agreement — 90.0% — which suggests variation may be due to changes in the syntax of outputs as a result of model stochasticity, updates to the internal weights or slight updates to the ontologies used for grounding.

## Discussion

Using a sample of 80 articles, we compared the agreement between OntoGPT and traditional manual extraction of 11 study attributes using two methods of evaluation: (1) manually comparing outputs and assigning agreement scores to each attribute, and (2) calculations of performance metrics (precision, recall, accuracy, F1) using term matching. These two approaches allowed robust validation and testing of OntoGPT’s capabilities to aid in extracting information from ecological literature on coastal wetland restoration outcomes and highlights the performance of a new method for automating the data extraction phase of an ecological evidence synthesis.

From our performance metric validation, we were able to glean additional insight into OntoGPT’s capabilities and assess the completeness of its data extractions (recall), and how accurate the data it extracted was (precision) [9]. The overall pattern in our results was similar to our manual agreement assessment, where we found higher performance metrics for latitude/longitude and date-based attributes compared to more challenging fields like sampling and monitoring methods. In general, our results indicate OntoGPT struggled to reliably extract information compared to the human reviewer baseline (macro-averages: precision 58%, recall 57%, accuracy 44%, F1 score 56%). We found that OntoGPT extractions had high proportions of FN (0.47) and FP (0.25) outcomes, leading to poor recall and precision, respectively. Its responses tended to contain extractions which were often not relevant, in line with a previous study by Spillias et al. [42], who found several different LLMs tended to extract excess information from literature on community-based fisheries management. Keck et al. [43] found excellent precision performance (90%) from LLM extraction of biological interactions from a large dataset but required post-extraction filtering to remove a large quantity of FN extractions. It is worth noting that our validation results are likely conservative estimates of model performance, as fuzzy matching of term-based attributes did not account for semantic overlap between synonymous terms with dissimilar character structures (e.g. grassland and prairie). For example, by incorporating a synonym dictionary to match just three terms, we noted increases in precision and recall for ecosystem types of 9% and 10%, respectively. Synonyms would be considered by OntoGPT if they were listed in the ontology, which points to the importance of improving the quality of ontologies to enhance term extraction. Similar to post-extraction filtering [43], this step improved model performance, but represents a trade-off between ease of implementation and post-processing effort.

From our agreement score results, we found that OntoGPT achieved 65% overall agreement with human annotators. However, agreement scores varied across attributes and were lowest on complex attributes. Our findings were in line with previous studies [44], where simpler attributes with information presented in a standardized form had higher agreement, compared to more complex attributes, which often involved study specific information, methodological terminology, and required advanced reasoning to assess their appropriateness for extraction. One exception to this pattern was restoration end date, a seemingly simple attribute that received an agreement score of only 50%. This was likely because restoration dates were generally complicated by multiple restoration actions occurring at different times, having poorly defined endpoints, or not being reported. Nevertheless, our findings suggest that OntoGPT could be helpful for extracting standard-format data that requires minimal interpretation or conversion. Limiting conversion of quantitative data may improve extraction performance, as we found 94% agreement for latitude/longitude when prompting OntoGPT to provide geographic coordinates in decimal degrees, degree minutes seconds, or any valid geospatial format. In contrast, Gougherty & Clipp [45], reported that 52% of LLM-extracted latitude/longitude coordinates from ecological literature had some degree of mismatch with human extractions due to conversion to decimal degree format. Future implementations of OntoGPT in ecological evidence syntheses could consider a combined approach, in which the tool rapidly extracts data for attributes that do not require interpretation, while human reviewers extract data on more nuanced, subjective attributes. Alternatively, a “human in the loop” approach could involve tasking OntoGPT with extracting information first, then having human reviewers verify and augment the extraction.

OntoGPT extracted more attribute entries per source literature item than reviewers (Table 6). This higher extraction rate can partially be attributed to repetition of reported taxa and the erroneous inclusion of non-focal species mentioned in the source literature. The over-extraction of information may present a challenge in syntheses as authors will have to verify which are the most relevant terms. This discrepancy was likely due to how OntoGPT outputs information. OntoGPT tended to extract single-word terms, whereas manual reviewers were more likely to identify multi-word terms and phrases. For example, manual reviewers extracted “soil sampling” and “invertebrate sampling” under the sampling/monitoring methods attribute, while OntoGPT extracted individual terms like “soil”, “invertebrate”, and “sampling”. The disparity between single-word and multi-word extractions was especially evident for complex attributes. This pattern highlights inherent problems in OntoGPT’s capacity to interpret and connect concepts together, and its reliance on single term keywords to extract information – possibly a function of its ontology grounding.

Situating our findings in the context of conservation and restoration science, a key feature of the field is that solutions are often context dependent [46], and there is an inherent complexity with studies spanning a wide array of ecosystem types and methods [47]. Accurate identification of complex attributes such as restoration actions, monitoring methods, and response variables can provide more relevant evidence to practitioners compared with simpler, but important, attributes like longitude/latitude or focal species [2, 48, 49]. , but current ontologies don’t contain sufficient information about these factors, and we found poor model performance in these important fields of high value to researchers.

### Limitations

This study used manual and automated approaches to compare the OntoGPT extraction with human reviewers. The manual comparison of agreement scores often required some interpretation based on the reviewers’ knowledge of the text, which may have led us to unintentionally favor ‘partial’ agreement, whereas a reviewer with no prior knowledge of the source literature may have deemed the extracted data to be in ‘disagreement’. However, our automated comparison (Sect. "Precision, recall, accuracy, and F1 score") provides an assessment not subject to human bias, though there are other considerations that may reflect the accuracy of that comparison (Sect. "Comparison of manual and OntoGPT data extraction"). Spillias et al. [42] also considered the issue of reviewer bias during extraction evaluation, but like us, opted for a non-blind approach to maintain a human standard for assessment. Similarly, we acknowledge that manual extraction has limitations and is susceptible to biases. Human error can occur, and reviewers can either miss key information or be inaccurate in extraction [50], potentially due to cognitive fatigue or differences in interpretation [51]. In cases where the human extractor was incorrect and OntoGPT was correct, it would still have registered as disagreement, potentially downplaying the accuracy of OntoGPT. We did not conduct a formal consistency check for the human extractors, though there was some comparison and discussion. A rigorous consistency check would increase the reliability of human extractions.

The reliance on ontologies provides the core functionality of OntoGPT, but may also be a technological limitation. Most ontologies are managed by volunteers and some are infrequently updated. Synonyms listed in ontologies may not be exhaustive, which could limit their usefulness for extracting text. Additionally, the use of PDF files for the source literature may have led to reduced accuracy as horizontal tables and images would not be subject to OntoGPT extraction. Finally, from a technological perspective, this study was conducted using GPT-4o. Newer models have been released which may perform differently than GPT-4o.

### Future directions

Based on our findings, there are several key items to be considered to improve the effectiveness of OntoGPT for environmental data extraction. Firstly, we advocate for the ongoing enhancement of ontologies, which are increasingly highlighted as reliable, expert-guided sources of knowledge [12]. In particular, the addition of synonyms in a rigorous, systematic and comprehensive way could greatly improve text extraction. Further expansion of the terms included in environmental ontologies will enhance OntoGPT’s ability to extract complex information by giving it greater capabilities to interpret and understand relationships between attribute-specific vocabularies and terms in the restoration literature. Additionally, frequently-updated structured data sources like Wikidata should be considered for grounding terms with large vocabularies that are frequently updated (e.g. taxa and locations) [52]. The concepts included in ontologies also do not adequately reflect ecological research. For instance, major ecosystem classification schemes are not included in ontologies [53, 54]. Ontologies would also benefit from a better depiction of restoration actions, as well as monitoring methods. We found that OntoGPT relied heavily on ENVTHES for its extraction. This is in part due to ENVTHES being assigned to more attributes in the prompt design, but it also suggests that this ontology has the most terms related to this field of research, and terms that are close to how authors write about the field. Other ontologies, such as ENVO, SWEET and PDO_CAS were underutilized and showed higher variability in extraction. This may be due to infrequent ontology updating (e.g., GAZ was last updated in 2015). The robustness of these and other environmental ontologies could be enhanced further with more frequent updates and the addition of new terminologies to ground extractions in the current state of the field. To this point, LLM-extraction evaluations such as ours could be used to identify relevant ecological terms that are not being returned from existing ontologies by inspecting FP and FN outcomes (e.g., Additional file 8). These are actionable ways for ecology researchers to contribute towards the effectiveness of AI as an environmental evidence synthesis tool. Another avenue for improving OntoGPT performance would be to invest more time into refining prompts. Schmidt et al. (2024) utilized several refinement strategies, including minor changes in wording, changes in the position sequence of prompts, and changes to field labels. Other studies have employed prompt engineering strategies such as “adopting a persona”, “chain of thought”, “few shot learning” or “Retrieval-Augmented Generation (RAG)” strategies to improve prompt efficiency and LLM accuracy in data extraction [55, 56]. Future directions for improvement of OntoGPT may involve some or all these strategies to increase overall performance across attributes. Effective prompt development and engineering is a critical component of LLM-assisted research and can be a challenge for non-experts [57], and as such, we encourage prospective researchers to budget appropriate time for thorough, iterative, prompt development [58].

Our selection of extraction fields was based on a systematic scoping review of Canadian ecological restoration research and derived from discussion among the co-authors [59]. The fields we chose reflect data types that are commonly found in restoration research: dates (e.g. restoration start date), taxa (e.g. focal species), ecosystems (e.g. ecosystem type), geospatial information (e.g. study site) and restoration actions. While we believe that our results are generalizable among other data types, it may be useful to conduct a more rigorous study of data types not analyzed in this study, such as fine measurements like soil moisture. This also provides important context for interpreting these results, as they may not hold if different data types are needed for the information extraction. Since our extraction was focused on data items appropriate for a scoping review, our results may not apply to a meta-analysis, where the data extracted is much more quantitative in nature and tends to come from tables and figures rather than the text.

Overall, our study demonstrates that OntoGPT holds potential as a methodological tool to assist with systematic reviews but requires human oversight. OntoGPT was most effective when extracting simpler attributes compared to more complex concepts and may result in time savings in that portion of the review. OntoGPT struggled to extract nuanced information like the methods of restoration and the response variables that define outcomes of restoration, limiting its utility to directly extract such attributes. However, it may be effective in augmenting human extraction, particularly for straightforward information like study location.

## Supplementary Information

Below is the link to the electronic supplementary material.

Supplementary Material 1.

Supplementary Material 2.

Supplementary Material 3.

Supplementary Material 4.

Supplementary Material 5.

Supplementary Material 6.

Supplementary Material 7.

Supplementary Material 8.

Supplementary Material 9.

Supplementary Material 10.

Supplementary Material 11.

## Data Availability

All data generated or analyzed during this study are included in this published article [and its supplementary information files].
