# Supplementary material for: Assessing the effectiveness of ontology-grounded AI term extraction using OntoGPT for environmental evidence synthesis
Source: Environ Evid. 2026 Feb 8;15:1. doi: 10.1186/s13750-026-00381-0 (PMC12892472; doi:10.1186/s13750-026-00381-0)
Supplement: Supplementary file 1 — Supplementary Material 1. [file 13750_2026_381_MOESM1_ESM.docx]

**Table A1.** Search string queries and number of items returned.

| **Source** | **Search String** | **Results** |
| --- | --- | --- |
| Web of Science | (TS=("habitat" OR "environment" OR "eco*") AND TS=("restoration" OR "regeneration" OR "rewild*" OR "re-wild*" OR "rehabilitation" OR "remediation" OR "rewet*" OR "re-wet*" OR "reclamation") AND TS=("success" OR "failure" OR "monitoring" OR "biomonitoring" OR "recovery" OR "status report" ) AND TS=("saltmarsh*" OR “salt marsh*” OR "mangrove*" OR “tidal marsh*” OR “tidal wetland*”) AND PY=(2009-2024)) NOT (SILOID==("PPRN")) | 1472 |
| Scopus | ( TITLE-ABS-KEY ( habitat OR "environment" OR "eco*" ) AND TITLE-ABS-KEY ( restoration OR "regeneration" OR "rewild*" OR "re-wild*" OR "rehabilitation" OR "remediation" OR "rewet*" OR "re-wet*" OR "reclamation" ) AND TITLE-ABS-KEY ( success OR "failure" OR "monitoring" OR "biomonitoring" OR "recovery" OR "status report") AND TITLE-ABS-KEY ( saltmarsh* OR "salt marsh*" OR "mangrove*" OR "tidal marsh*" OR "tidal wetland*" ) ) AND PUBYEAR > 2008 | 1100 |
| Google Scholar | ("habitat" OR "environment" OR "eco*") AND ("restoration" OR "regeneration" OR "rewild*" OR "re-wild*" OR "rehabilitation" OR "remediation" OR "rewet*" OR "re-wet*" OR "reclamation") AND ("success" OR "failure" OR "monitoring" OR "biomonitoring" OR "recovery" OR "status report") AND ("saltmarsh*" OR “salt marsh*” OR "mangrove*" OR “tidal marsh*” OR “tidal wetland*”) | 200 |
