# Supplementary material for: Assessing the effectiveness of ontology-grounded AI term extraction using OntoGPT for environmental evidence synthesis
Source: Environ Evid. 2026 Feb 8;15:1. doi: 10.1186/s13750-026-00381-0 (PMC12892472; doi:10.1186/s13750-026-00381-0)
Supplement: Supplementary file 2 — Supplementary Material 2. [file 13750_2026_381_MOESM2_ESM.docx]

**Example Extraction Agreement Assessments**

| **Table 1.** Agreement assessment between manual and OntoGPT data extractions for attributes in Bashan et al. (2014). An agreement score was assigned for each attribute: 2 = agreement, 1 = partial agreement, and 0 = disagreement. | | | |
| --- | --- | --- | --- |
| **Attribute** | **Manual Extraction** | **OntoGPT Extraction** | **Agreement Score** |
| Study Site | La Paz, Baja, California Sur, Mexico | La Paz | 1 |
| Latitude/longitude | 24 08'N, 110 23'W | 24°08'N, 110°23'W | 2 |
| Ecosystem Type | Mangrove, lagoon | Mangrove, marine | 1 |
| Restoration Actions | low knickpoint/waterfall creation, hydrologic change to remove sediments, Hydrologic restoration | Creating a small waterfall, restoration, flow | 2 |
| Restoration Start date | 2004-04 | 4/1/2004 | 2 |
| Restoration End date | 2004-06 | 6/30/2004 | 2 |
| Sampling/monitoring methods | Satellite imagery, vegetation inventory | Satellite, global positioning system, field observations, photographic records, vegetation, sediment, water turbidity of stagnant surface water | 1 |
| Monitoring Start Date | 2007 | 2/1/2009 | 0 |
| Monitoring End date | 2012 | 7/6/2011 | 0 |
| Focal Species | Rhizophora mangle L., Languncularia racemosa Gaertn., Avicennia germinans (L.) | Rhizophora mangle, Laguncularia racemosa, Avicennia, Salicornia bigelovii | 1 |
| Response variables | Hydrologic function, Hydraulic connectivity, Net sediment transport, Tree coverage, mangrove area, % tree coverage by species, tidal flow (+) | Vegetation cover, sediment transport, sediment | 1 |

| **Table 2.** Agreement assessment between manual and OntoGPT data extractions for attributes in Jiang et al. (2024). An agreement score was assigned for each attribute: 2 = agreement, 1 = partial agreement, and 0 = disagreement. | | | |
| --- | --- | --- | --- |
| **Attribute** | **Manual Extraction** | **OntoGPT Extraction** | **Agreement Score** |
| Study Site | Dongtan Wetlands, Chongming Island, Yangtze Estuary | Chongming Island Wetland | 2 |
| Latitude/longitude | 31°27'–31°51' N, 121°09'–121°54' E | 31°270–31°510 N, 121°090–121°540 E | 2 |
| Ecosystem Type | Salt marsh | Salt marsh | 2 |
| Restoration Actions | native phragmites planting, invasive spartina removal | Spatina removal, phragmites transplanting | 2 |
| Restoration Start date | none reported | 2012 | 0 |
| Restoration End date | none reported | 2017 | 0 |
| Sampling/monitoring methods | transect sampling, arthropod sampling, vegetation sampling, stable isotopes, soil sampling | Vacuum suctioning | 0 |
| Monitoring Start Date | None reported | 6/22/2018 | 0 |
| Monitoring End date | None reported | 8/26/2018 | 0 |
| Focal Species | Spartina alterniflora, Phragmites australis | Sporobolus alterniflorus, Phragmites | 2 |
| Response variables | species richness, individual density, feeding guild abundance, N, C, stable isotope signatures, biomass, plant density, leaf N, soil salinity | Diversity, community structure, Composition, natural | 1 |
