# Supplementary material for: Assessing the effectiveness of ontology-grounded AI term extraction using OntoGPT for environmental evidence synthesis
Source: Environ Evid. 2026 Feb 8;15:1. doi: 10.1186/s13750-026-00381-0 (PMC12892472; doi:10.1186/s13750-026-00381-0)
Supplement: Supplementary file 3 — Supplementary Material 3. [file 13750_2026_381_MOESM3_ESM.docx]

**Table A2**. Inclusion criteria used for title and abstract screening.

| **Criteria** | **Definition for Inclusion** |
| --- | --- |
| Language | Include only studies published in English |
| Ecosystem type | Focus on papers specifically addressing coastal wetlands (e.g., salt marshes, mangroves, estuaries). Exclude inland or freshwater wetlands. |
| Article type | Include primary peer reviewed research only. Exclude meta-analyses, systematic reviews, modeling papers, opinion papers, news articles, graduate dissertations, policy briefs and book chapters. |
| Study type | Only include field research studies that discuss an outcome after a restoration effort. Exclude all greenhouse or other laboratory-based studies. All studies should include a study site that is either restored or where restoration is underway. |
