# Supplementary material for: Assessing the effectiveness of ontology-grounded AI term extraction using OntoGPT for environmental evidence synthesis
Source: Environ Evid. 2026 Feb 8;15:1. doi: 10.1186/s13750-026-00381-0 (PMC12892472; doi:10.1186/s13750-026-00381-0)
Supplement: Supplementary file 4 — Supplementary Material 4. [file 13750_2026_381_MOESM4_ESM.docx]

**Source Literature Reference List**

Abbott, B. N., Wallace, J., Nicholas, D. M., Karim, F., & Waltham, N. J. (2020). Bund removal to re-establish tidal flow, remove aquatic weeds and restore coastal wetland services—North Queensland, Australia. *PLOS ONE*, *15*(1), e0217531. <https://doi.org/10.1371/journal.pone.0217531>

Adgie, T. E., & Chapman, S. K. (2021). Salt marsh plant community structure influences success of *Avicennia germinans* during poleward encroachment. *Wetlands*, *41*(6), 82. <https://doi.org/10.1007/s13157-021-01463-0>

Al-Khayat, J. A., Abdulla, M. A., & Alatalo, J. M. (2019). Diversity of benthic macrofauna and physical parameters of sediments in natural mangroves and in afforested mangroves three decades after compensatory planting. *Aquatic Sciences*, *81*(1), 4. <https://doi.org/10.1007/s00027-018-0599-7>

Amelia, R., Basyuni, M., Alfinsyahri, A., Sulistiyono, N., Slamet, B., Bimantara, Y., Harahap, S. S. H., Harahap, M., Harahap, I. M., Al Mustaniroh, S. S., Sasmito, S. D., & Arifanti, V. B. (2023). Evaluation of plant growth and potential of carbon Ssorage in the restored Mangrove of an abandoned pond in Lubuk Kertang, North Sumatra, Indonesia. *Forests*, *14*(1), 158. <https://doi.org/10.3390/f14010158>

Ashton, E. C., & Macintosh, D. J. (2024). Mangrove rehabilitation and brachyuran crab biodiversity in Ranong, Thailand. *Diversity*, *16*(2), 92. <https://doi.org/10.3390/d16020092>

Aye, W. M., & Takeda, S. (2020). Conversion of abandoned paddy fields to productive land through mangrove restoration in Myanmar’s Ayeyarwady Delta. *Paddy and Water Environment*, *18*(2), 417–429. <https://doi.org/10.1007/s10333-020-00791-x>

Bakrin Sofawi, A., Rozainah, M. Z., Normaniza, O., & Roslan, H. (2017). Mangrove rehabilitation on Carey Island, Malaysia: An evaluation of replanting techniques and sediment properties. *Marine Biology Research*, *13*(4), 390–401. <https://doi.org/10.1080/17451000.2016.1267365>

Bashan, Y., Moreno, M., Salazar, B. G., & Alvarez, L. (2013). Restoration and recovery of hurricane-damaged mangroves using the knickpoint retreat effect and tides as dredging tools. *Journal of Environmental Management*, *116*, 196–203. <https://doi.org/10.1016/j.jenvman.2012.11.045>

Beheshti, K. M., Schroeter, S. C., Deza, A. A., Reed, D. C., Smith, R. S., & Page, H. M. (2023). Large‐scale field studies inform adaptive management of California wetland restoration. *Restoration Ecology*, *31*(5), e13936. <https://doi.org/10.1111/rec.13936>

Bernhard, A. E., Dwyer, C., Idrizi, A., Bender, G., & Zwick, R. (2015). Long-term impacts of disturbance on nitrogen-cycling bacteria in a New England salt marsh. *Frontiers in Microbiology*, *6*. <https://doi.org/10.3389/fmicb.2015.00046>

Boys, C. A., Kroon, F. J., Glasby, T. M., & Wilkinson, K. (2012). Improved fish and crustacean passage in tidal creeks following floodgate remediation. *Journal of Applied Ecology*, *49*(1), 223–233. <https://doi.org/10.1111/j.1365-2664.2011.02101.x>

Carneiro, I., Carrasco, A. R., Didderen, K., & Sousa, A. I. (2024). Evaluating the success of vegetation restoration in rewilded salt marshes. *Science of The Total Environment*, *926*, 171699. <https://doi.org/10.1016/j.scitotenv.2024.171699>

Chang, E. R., Veeneklaas, R. M., Bakker, J. P., Daniels, P., & Esselink, P. (2016). What factors determined restoration success of a salt marsh ten years after de‐embankment? *Applied Vegetation Science*, *19*(1), 66–77. <https://doi.org/10.1111/avsc.12195>

Charoenlerkthawin, W., Bidorn, K., Burnett, W. C., Sopon, A., Otarawanna, S., & Bidorn, B. (2024). Evaluating the effectiveness of mangrove rehabilitation: A novel approach for sustainable coastal management. *Journal of Environmental Management*, *365*, 121673. <https://doi.org/10.1016/j.jenvman.2024.121673>

Chen, G., Bai, J., Yu, L., Chen, B., Zhang, Y., Liu, G., & Wang, W. (2022). Effects of ecological restoration on carbon sink and carbon drawdown of degraded salt marshes with carbon‐rich additives application. *Land Degradation & Development*, *33*(12), 2103–2114. <https://doi.org/10.1002/ldr.4306>

Chu, T.-J., Shih, Y.-J., Shih, C.-H., Wang, J.-Q., Huang, L.-M., & Tsai, S.-C. (2022). Developing a model to select indicator species based on individual species’ contributions to biodiversity. *Applied Sciences*, *12*(13), 6748. <https://doi.org/10.3390/app12136748>

Curado, G., Manzano-Arrondo, V., Figueroa, E., & Castillo, j. M. (2014). Public perceptions and uses of natural and restored salt marshes. *Landscape Research*, *39*(6), 668–679. <https://doi.org/10.1080/01426397.2013.772960>

Damastuti, E., De Groot, R., Debrot, A. O., & Silvius, M. J. (2022). Effectiveness of community-based mangrove management for biodiversity conservation: A case study from Central Java, Indonesia. *Trees, Forests and People*, *7*, 100202. <https://doi.org/10.1016/j.tfp.2022.100202>

Davis, M. J., Woo, I., & De La Cruz, S. E. W. (2019). Development and implementation of an empirical habitat change model and decision support tool for estuarine ecosystems. *Ecological Modelling*, *410*, 108722. <https://doi.org/10.1016/j.ecolmodel.2019.108722>

Derksen‐Hooijberg, M., Angelini, C., Lamers, L. P. M., Borst, A., Smolders, A., Hoogveld, J. R. H., De Paoli, H., Van De Koppel, J., Silliman, B. R., & Van Der Heide, T. (2018). Mutualistic interactions amplify saltmarsh restoration success. *Journal of Applied Ecology*, *55*(1), 405–414. <https://doi.org/10.1111/1365-2664.12960>

Dibble, K. L., & Meyerson, L. A. (2012). Tidal flushing restores the physiological condition of fish residing in degraded salt marshes. *PLoS ONE*, *7*(9), e46161. <https://doi.org/10.1371/journal.pone.0046161>

Evens, J. (2020). Temporal response of California Black Rails to tidal wetland restoration. *Western Birds*, *51*(2), 111–121. <https://doi.org/10.21199/WB51.2.4>

Farrugia, T. J., Espinoza, M., & Lowe, C. G. (2014). The fish community of a newly restored southern California estuary: Ecological perspective 3 years after restoration. *Environmental Biology of Fishes*, *97*(10), 1129–1147. <https://doi.org/10.1007/s10641-013-0203-x>

Green, J., Reichelt‐Brushett, A., & Jacobs, S. W. L. (2009). Re‐establishing a saltmarsh vegetation structure in a changing climate. *Ecological Management & Restoration*, *10*(1), 20–30. <https://doi.org/10.1111/j.1442-8903.2009.00438.x>

Guo, P., Lin, Y., Sheng, Y., Gu, X., Deng, Y., Zhang, Y., Wang, W., & Wang, M. (2024). Comparison of the coexistence pattern of mangrove macrobenthos between natural and artificial reforestation. *Ecology and Evolution*, *14*(8), e70069. <https://doi.org/10.1002/ece3.70069>

Hernández‐Carrasco, D., Cunillera‐Montcusí, D., Antón‐Pardo, M., Cañedo‐Argüelles, M., Bas‐Silvestre, M., Compte, J., Gascón, S., Quintana, X. D., & Boix, D. (2023). Ecological restoration promotes zooplankton network complexity in Mediterranean coastal lagoons. *Restoration Ecology*, *31*(5), e13920. <https://doi.org/10.1111/rec.13920>

Hu, Z.-J., Ge, Z.-M., Ma, Q., Zhang, Z.-T., Tang, C.-D., Cao, H.-B., Zhang, T.-Y., Li, B., & Zhang, L.-Q. (2015). Revegetation of a native species in a newly formed tidal marsh under varying hydrological conditions and planting densities in the Yangtze Estuary. *Ecological Engineering*, *83*, 354–363. <https://doi.org/10.1016/j.ecoleng.2015.07.005>

Hua, Y., Cui, B., & He, W. (2012). Changes in water birds habitat suitability following wetland restoration in the Yellow River Delta, China. *CLEAN – Soil, Air, Water*, *40*(10), 1076–1084. <https://doi.org/10.1002/clen.201200064>

Jiang, J., Zhao, Y., Guo, Y., Gao, L., Richards, C. L., Siemann, E., Wu, J., Li, B., & Ju, R. (2024). Restoration of native saltmarshes can reverse arthropod assemblages and trophic interactions changed by a plant invasion. *Ecological Applications*, *34*(1), e2740. <https://doi.org/10.1002/eap.2740>

Jimenez, L. C. Z., Queiroz, H. M., Otero, X. L., Nóbrega, G. N., & Ferreira, T. O. (2021). Soil organic matter responses to mangrove restoration: A replanting experience in northeast Brazil. *International Journal of Environmental Research and Public Health*, *18*(17), 8981. <https://doi.org/10.3390/ijerph18178981>

Josephs, L. I., & Humphries, A. T. (2018). Identifying social factors that undermine support for nature-based coastal management. *Journal of Environmental Management*, *212*, 32–38. <https://doi.org/10.1016/j.jenvman.2018.01.085>

Kinya, G., Kairo, J. G., Nyoike, R. N., Nguu, J. G., Githinji, B. K., & Githaiga, M. N. (2024). Eco-engineering mangrove restoration at Gazi Bay, Kenya. *Diversity*, *16*(3), 135. <https://doi.org/10.3390/d16030135>

Koo, B. J., Je, J. G., & Woo, H. J. (2011). Experimental restoration of a salt marsh with some comments on ecological restoration of coastal vegetated ecosystems in Korea. *Ocean Science Journal*, *46*(1), 47–53. <https://doi.org/10.1007/s12601-011-0004-0>

Lawrence, P. J., Sullivan, M. J. P., & Mossman, H. L. (2022). Restored saltmarshes have low beta diversity due to limited topographic variation, but this can be countered by management. *Journal of Applied Ecology*, *59*(7), 1709–1720. <https://doi.org/10.1111/1365-2664.14179>

Loch, J. M. H., & Cook, G. S. (2023). Evidence of ontogenetic partitioning of restored coastal habitat by a generalist sportfish. *Restoration Ecology*, *31*(7), e13960. <https://doi.org/10.1111/rec.13960>

Lynum, C. A., Bulseco, A. N., Dunphy, C. M., Osborne, S. M., Vineis, J. H., & Bowen, J. L. (2020). Microbial community response to a passive salt marsh restoration. *Estuaries and Coasts*, *43*(6), 1439–1455. <https://doi.org/10.1007/s12237-020-00719-y>

Mahoney, R. D., Beal, J. L., Lewis, D. M., & Cook, G. S. (2021). Quantifying the response of an estuarine nekton community to coastal wetland habitat restoration. *Sustainability*, *13*(23), 13299. <https://doi.org/10.3390/su132313299>

Manley, J., Power, A., Walker, R., Hurley, D., Belcher, C., & Richardson, J. (2010). Ecological succession on restored intertidal oyster habitat in the tidal creeks of coastal Georgia. *Journal of Shellfish Research*, *29*(4), 917–926. <https://doi.org/10.2983/035.029.0424>

Masselink, G., Hanley, M. E., Halwyn, A. C., Blake, W., Kingston, K., Newton, T., & Williams, M. (2017). Evaluation of salt marsh restoration by means of self-regulating tidal gate – Avon estuary, South Devon, UK. *Ecological Engineering*, *106*, 174–190. <https://doi.org/10.1016/j.ecoleng.2017.05.038>

Matsui, N., Suekuni, J., Nogami, M., Havanond, S., & Salikul, P. (2010). Mangrove rehabilitation dynamics and soil organic carbon changes as a result of full hydraulic restoration and re-grading of a previously intensively managed shrimp pond. *Wetlands Ecology and Management*, *18*(2), 233–242. <https://doi.org/10.1007/s11273-009-9162-6>

Milbrandt, E. C., Thompson, M., Coen, L. D., Grizzle, R. E., & Ward, K. (2015). A multiple habitat restoration strategy in a semi-enclosed Florida embayment, combining hydrologic restoration, mangrove propagule plantings and oyster substrate additions. *Ecological Engineering*, *83*, 394–404. <https://doi.org/10.1016/j.ecoleng.2015.06.043>

Nguyen, T. P., Van Tam, N., Quoi, L. P., & Parnell, K. E. (2016). Community perspectives on an internationally funded mangrove restoration project: Kien Giang province, Vietnam. *Ocean & Coastal Management*, *119*, 146–154. <https://doi.org/10.1016/j.ocecoaman.2015.10.008>

Noel, P. E., Sharma, B., & Chmura, G. L. (2023). Invertebrate communities of Bay of Fundy salt marsh pools: Comparison of a natural and recovering marsh. *Frontiers in Ecology and Evolution*, *11*, 994533. <https://doi.org/10.3389/fevo.2023.994533>

Norris, G. S., Virgin, S. D. S., Schneider, D. W., McCoy, E. M., Wilson, J. M., Morrill, K. L., Hayter, L., Hicks, M. E., & Barbeau, M. A. (2022). Patch-level processes of vegetation underlying site-level restoration patterns in a megatidal salt marsh. *Frontiers in Ecology and Evolution*, *10*, 1000075. <https://doi.org/10.3389/fevo.2022.1000075>

Oh, R. R. Y., Friess, D. A., & Brown, B. M. (2017). The role of surface elevation in the rehabilitation of abandoned aquaculture ponds to mangrove forests, Sulawesi, Indonesia. *Ecological Engineering*, *100*, 325–334. <https://doi.org/10.1016/j.ecoleng.2016.12.021>

Pagliosa, P. R., Oortman, M. S., Rovai, A. S., & Soriano-Sierra, E. J. (2016). Is mangrove planting insufficient for benthic macrofaunal recovery when environmental stress is persistent? *Ecological Engineering*, *95*, 290–301. <https://doi.org/10.1016/j.ecoleng.2016.06.036>

Pausch, R. (2024). Testing strategies to enhance transplant success under stressful conditions at a tidal marsh restoration project. *Restoration Ecology*, *32*(4), e14117. <https://doi.org/10.1111/rec.14117>

Peng, Y., Diao, J., Zheng, M., Guan, D., Zhang, R., Chen, G., & Lee, S. Y. (2016). Early growth adaptability of four mangrove species under the canopy of an introduced mangrove plantation: Implications for restoration. *Forest Ecology and Management*, *373*, 179–188. <https://doi.org/10.1016/j.foreco.2016.04.044>

Pérez-Ceballos, R., Echeverría-Ávila, S., Zaldívar-Jiménez, A., Zaldívar-Jiménez, T., & Herrera-Silveira, J. (2017). Contribution of microtopography and hydroperiod to the natural regeneration of *Avicennia germinans* in a restored mangrove forest. *Ciencias Marinas*, *43*(1), 55–67. <https://doi.org/10.7773/cm.v43i1.2683>

Pétillon, J., Potier, S., Carpentier, A., & Garbutt, A. (2014). Evaluating the success of managed realignment for the restoration of salt marshes: Lessons from invertebrate communities. *Ecological Engineering*, *69*, 70–75. <https://doi.org/10.1016/j.ecoleng.2014.03.085>

Qian, W., Chen, J., Zhang, Q., Wu, C., Ma, Q., Silliman, B. R., Wu, J., Li, B., & He, Q. (2021). Top-down control of foundation species recovery during coastal wetland restoration. *Science of The Total Environment*, *769*, 144854. <https://doi.org/10.1016/j.scitotenv.2020.144854>

Qiu, D., Cui, B., Ma, X., Yan, J., Cai, Y., Xie, T., Gao, F., Wang, F., Sui, H., Bai, J., Van De Koppel, J., & Olff, H. (2021). Reciprocal facilitation between annual plants and burrowing crabs: Implications for the restoration of degraded saltmarshes. *Journal of Ecology*, *109*(4), 1828–1841. <https://doi.org/10.1111/1365-2745.13608>

Raposa, K. B., Bradley, M., Chaffee, C., Ernst, N., Ferguson, W., Kutcher, T. E., McKinney, R. A., Miller, K. M., Rasmussen, S., Tymkiw, E., & Wigand, C. (2022). Laying it on thick: Ecosystem effects of sediment placement on a microtidal Rhode Island salt marsh. *Frontiers in Environmental Science*, *10*, 939870. <https://doi.org/10.3389/fenvs.2022.939870>

Retnaningdyah, C., Cahya Febriansyah, S., & Hakim, L. (2023). Evaluation of the quality of mangrove ecosystems using macrozoobenthos as bioindicators in the Southern Coast of East Java, Indonesia. *Biodiversitas Journal of Biological Diversity*, *23*(12). <https://doi.org/10.13057/biodiv/d231247>

Rovai, A. S., Barufi, J. B., Pagliosa, P. R., Scherner, F., Torres, M. A., Horta, P. A., Simonassi, J. C., Quadros, D. P. C., Borges, D. L. G., & Soriano-Sierra, E. J. (2013). Photosynthetic performance of restored and natural mangroves under different environmental constraints. *Environmental Pollution*, *181*, 233–241. <https://doi.org/10.1016/j.envpol.2013.06.023>

Sánchez‐Núñez, D. A., Rodríguez‐Rodríguez, J. A., & Mancera Pineda, J. E. (2023). Effects of climate variability and hydrological rehabilitation measures on long‐term mangrove trajectories: From reproduction to recruitment and landscape cover changes. *Journal of Applied Ecology*, *60*(12), 2508–2520. <https://doi.org/10.1111/1365-2664.14536>

Santín, C., De La Rosa, J. M., Knicker, H., Otero, X. L., Álvarez, M. Á., & González-Vila, F. J. (2009). Effects of reclamation and regeneration processes on organic matter from estuarine soils and sediments. *Organic Geochemistry*, *40*(9), 931–941. <https://doi.org/10.1016/j.orggeochem.2009.06.005>

Santini, N. S., Lovelock, C. E., Hua, Q., Zawadzki, A., Mazumder, D., Mercer, T. R., Muñoz-Rojas, M., Hardwick, S. A., Madala, B. S., Cornwell, W., Thomas, T., Marzinelli, E. M., Adam, P., Paul, S., & Vergés, A. (2019). Natural and regenerated saltmarshes exhibit similar soil and belowground organic carbon stocks, root production and soil respiration. *Ecosystems*, *22*(8), 1803–1822. <https://doi.org/10.1007/s10021-019-00373-x>

Slee, N. J. D., Gardiner, T., & Underwood, G. J. C. (2023). Hybrid engineering incorporating salt marsh terraces into sea wall repair maintains their defence function and creates new habitats. *Estuarine, Coastal and Shelf Science*, *294*, 108544. <https://doi.org/10.1016/j.ecss.2023.108544>

Stagg, C. L., & Mendelssohn, I. A. (2010). Restoring ecological function to a submerged salt marsh. *Restoration Ecology*, *18*(s1), 10–17. <https://doi.org/10.1111/j.1526-100X.2010.00718.x>

Stagg, C. L., & Mendelssohn, I. A. (2011). Controls on resilience and stability in a sediment-subsidized salt marsh. *Ecological Applications*, *21*(5), 1731–1744. <https://doi.org/10.1890/09-2128.1>

Staszak, L. A., & Armitage, A. R. (2013). Evaluating salt marsh restoration success with an index of ecosystem integrity. *Journal of Coastal Research*, *287*, 410–418. <https://doi.org/10.2112/JCOASTRES-D-12-00075.1>

Sullivan, M. J. P., Davy, A. J., Grant, A., & Mossman, H. L. (2018). Is saltmarsh restoration success constrained by matching natural environments or altered succession? A test using niche models. *Journal of Applied Ecology*, *55*(3), 1207–1217. <https://doi.org/10.1111/1365-2664.13033>

Sulochanan, B., Ratheesh, L., Veena, S., Padua, S., Prema, D., Rohit, P., Kaladharan, P., & Kripa, V. (2022). Water and sediment quality parameters of the restored mangrove ecosystem of Gurupura River and natural mangrove ecosystem of Shambhavi River in Dakshina Kannada, India. *Marine Pollution Bulletin*, *176*, 113450. <https://doi.org/10.1016/j.marpolbul.2022.113450>

Tackley, H. A., Kurylyk, B. L., Lake, C. B., Lapen, D. R., & Van Proosdij, D. (2023). Impacts of repeated coastal flooding on soil and groundwater following managed dike realignment. *Science of The Total Environment*, *893*, 164957. <https://doi.org/10.1016/j.scitotenv.2023.164957>

Temmink, R. J. M., Christianen, M. J. A., Fivash, G. S., Angelini, C., Boström, C., Didderen, K., Engel, S. M., Esteban, N., Gaeckle, J. L., Gagnon, K., Govers, L. L., Infantes, E., Van Katwijk, M. M., Kipson, S., Lamers, L. P. M., Lengkeek, W., Silliman, B. R., Van Tussenbroek, B. I., Unsworth, R. K. F., … Van Der Heide, T. (2020). Mimicry of emergent traits amplifies coastal restoration success. *Nature Communications*, *11*(1), 3668. <https://doi.org/10.1038/s41467-020-17438-4>

Thiet, R. K., Kidd, E., Wennemer, J. M., & Smith, S. M. (2014). Molluscan community recovery in a New England back‐barrier salt marsh lagoon 10 years after partial restoration. *Restoration Ecology*, *22*(4), 447–455. <https://doi.org/10.1111/rec.12083>

Thornton, S. R., & Johnstone, R. W. (2015). Mangrove rehabilitation in high erosion areas: Assessment using bioindicators. *Estuarine, Coastal and Shelf Science*, *165*, 176–184. <https://doi.org/10.1016/j.ecss.2015.05.013>

Van Bijsterveldt, C. E. J., Debrot, A. O., Bouma, T. J., Maulana, M. B., Pribadi, R., Schop, J., Tonneijck, F. H., & Van Wesenbeeck, B. K. (2022). To plant or not to plant: When can planting facilitate mangrove restoration? *Frontiers in Environmental Science*, *9*, 690011. <https://doi.org/10.3389/fenvs.2021.690011>

Van Der Heide, T., Temmink, R. J. M., Fivash, G. S., Bouma, T. J., Boström, C., Didderen, K., Esteban, N., Gaeckle, J., Gagnon, K., Infantes, E., Van De Koppel, J., Lengkeek, W., Unsworth, R., & Christianen, M. J. A. (2021). Coastal restoration success via emergent trait-mimicry is context dependent. *Biological Conservation*, *264*, 109373. <https://doi.org/10.1016/j.biocon.2021.109373>

Van Proosdij, D., Lundholm, J., Neatt, N., Bowron, T., & Graham, J. (2010). Ecological re-engineering of a freshwater impoundment for salt marsh restoration in a hypertidal system. *Ecological Engineering*, *36*(10), 1314–1332. <https://doi.org/10.1016/j.ecoleng.2010.06.008>

Veenklaas, R. M., Koppenaal, E. C., Bakker, J. P., & Esselink, P. (2015). Salinization during salt-marsh restoration after managed realignment. *Journal of Coastal Conservation*, *19*(4), 405–415. <https://doi.org/10.1007/s11852-015-0390-z>

Vovides, A. G., Bashan, Y., López‐Portillo, J. A., & Guevara, R. (2011). Nitrogen fixation in preserved, reforested, naturally regenerated and impaired mangroves as an indicator of functional restoration in mangroves in an arid region of Mexico. *Restoration Ecology*, *19*(201), 236–244. <https://doi.org/10.1111/j.1526-100X.2010.00713.x>

Wang, Q., Cui, B., & Luo, M. (2018). Effectiveness of microtopographic structure in species recovery in degraded salt marshes. *Marine Pollution Bulletin*, *133*, 173–181. <https://doi.org/10.1016/j.marpolbul.2018.05.037>

Wang, Q., Cui, B., Luo, M., & Shi, W. (2018). Designing microtopographic structures to facilitate seedling recruitment in degraded salt marshes. *Ecological Engineering*, *120*, 266–273. <https://doi.org/10.1016/j.ecoleng.2018.06.012>

Wood, S. E., White, J. R., & Armbruster, C. K. (2017). Microbial processes linked to soil organic matter in a restored and natural coastal wetland in Barataria Bay, Louisiana. *Ecological Engineering*, *106*, 507–514. <https://doi.org/10.1016/j.ecoleng.2017.06.028>

Zhao, P., Sanganyado, E., Wang, T., Sun, Z., Jiang, Z., Zeng, M., Huang, Z., Li, Y., Li, P., Bi, R., & Liu, W. (2022). Accumulation of nutrients and potentially toxic elements in plants and fishes in restored mangrove ecosystems in South China. *Science of The Total Environment*, *838*, 155964. <https://doi.org/10.1016/j.scitotenv.2022.155964>

Zhao, Q., Bai, J., Gao, Y., Zhao, H., Huang, Y., Zhang, W., Wang, J., & Chen, G. (2019). Effects of freshwater inputs on soil quality in the Yellow River Delta, China. *Ecological Indicators*, *98*, 619–626. <https://doi.org/10.1016/j.ecolind.2018.11.041>

Zhao, X., Wang, C., Li, T., Zhang, C., Fan, X., Zhang, Q., Zhang, Q., Chen, X., Zou, X., Shen, C., Tang, Y., & Qin, Z. (2022). Net CO2 and CH4 emissions from restored mangrove wetland: New insights based on a case study in estuary of the Pearl River, China. *Science of The Total Environment*, *811*, 151619. <https://doi.org/10.1016/j.scitotenv.2021.151619>

Zhou, T., Liu, S., Feng, Z., Liu, G., Gan, Q., & Peng, S. (2015). Use of exotic plants to control *Spartina alterniflora* invasion and promote mangrove restoration. *Scientific Reports*, *5*(1), 12980. <https://doi.org/10.1038/srep12980>
