# Supplementary material for: Assessing the effectiveness of ontology-grounded AI term extraction using OntoGPT for environmental evidence synthesis
Source: Environ Evid. 2026 Feb 8;15:1. doi: 10.1186/s13750-026-00381-0 (PMC12892472; doi:10.1186/s13750-026-00381-0)
Supplement: Supplementary file 7 — Supplementary Material 7. [file 13750_2026_381_MOESM7_ESM.docx]

**Title and Abstract Screening**

Manual screening was carried out in Rayyan (Ouzzani et al., 2016) by AB, FC, RH and SR to include articles that met our inclusion/exclusion criteria (Table A2). This involved screening abstracts and titles, including only English peer-reviewed studies that discussed ecological restoration outcomes and used field-based research methods in coastal wetland environments.

We performed a consistency check to determine the level of agreement among reviewer screening decisions by simultaneously screening a random sample of 183 articles (10% of the 1,829 dataset) and comparing inclusion decisions. Inclusion/exclusion decisions were compared using Fleiss’ Kappa coefficient — a statistical measure of reliability of agreement between two or more raters (Fleiss, 1971). The resulting Fleiss’ Kappa score was *κ* = 0.57 (n = 183, p <0.001), indicating “moderate” agreement (Landis & Koch, 1977). There was unanimous inclusion/exclusion agreement for 69.9% of articles, with disagreements primarily resulting from one reviewer assigning a “maybe.” All disagreements were resolved through discussion leading to a unanimous screening decision for each publication. After the consistency check, remaining unscreened articles (n = 1,646) were divided among the four reviewers for title and abstract screening, yielding 496 papers for inclusion. See Figure A1 for a ROSES flow diagram of the literature search and screening process.


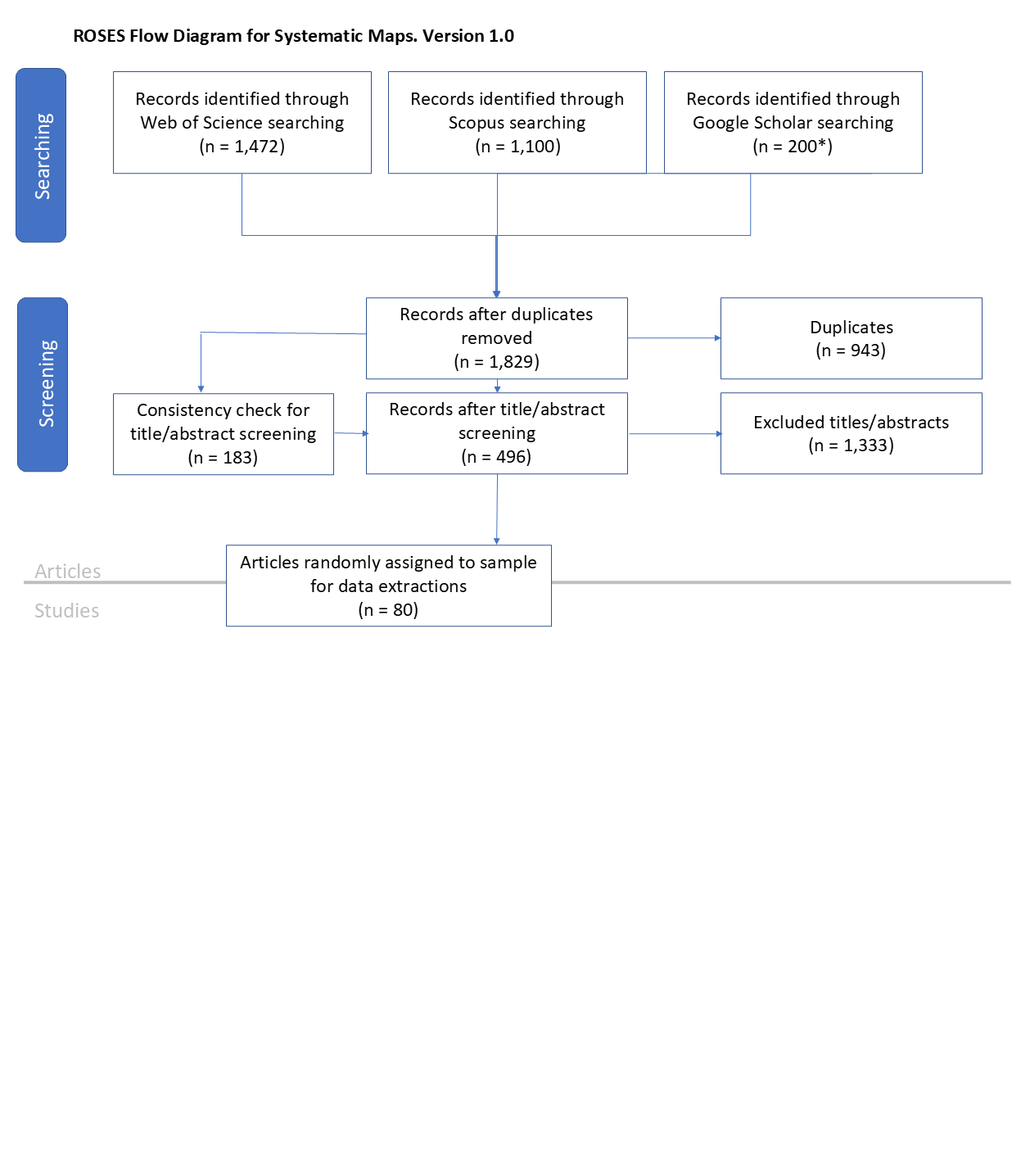


**Figure A1**. ROSES flow diagram of literature search and screening process.
