# Supplementary material for: Assessing the effectiveness of ontology-grounded AI term extraction using OntoGPT for environmental evidence synthesis
Source: Environ Evid. 2026 Feb 8;15:1. doi: 10.1186/s13750-026-00381-0 (PMC12892472; doi:10.1186/s13750-026-00381-0)
Supplement: Supplementary file 9 — Supplementary Material 9. [file 13750_2026_381_MOESM9_ESM.docx]

**Synopsis of Review Article Locations and Ecosystem Types**

Based on manually extracted data from a sample (n = 80) of coastal wetland restoration source literature, we found that most study sites were in Asia (35%), North America (32%), and Europe (18%), with the United States (n = 22) and China (n = 13) accounting for 43% of all locations (Figure A2). Countries with modest representation included the United Kingdom (n = 6), Indonesia (n = 5), Australia (n = 4) and Canada (n = 4). Africa was poorly represented, with a single study in Tanzania.

Ecosystem types were predominantly salt marsh (45%) or mangrove (28%). Publications that noted a combination of salt marsh, mangrove, and/or estuarine ecosystem types comprised 14% of the sample, while locations described only as estuaries represented 5%.


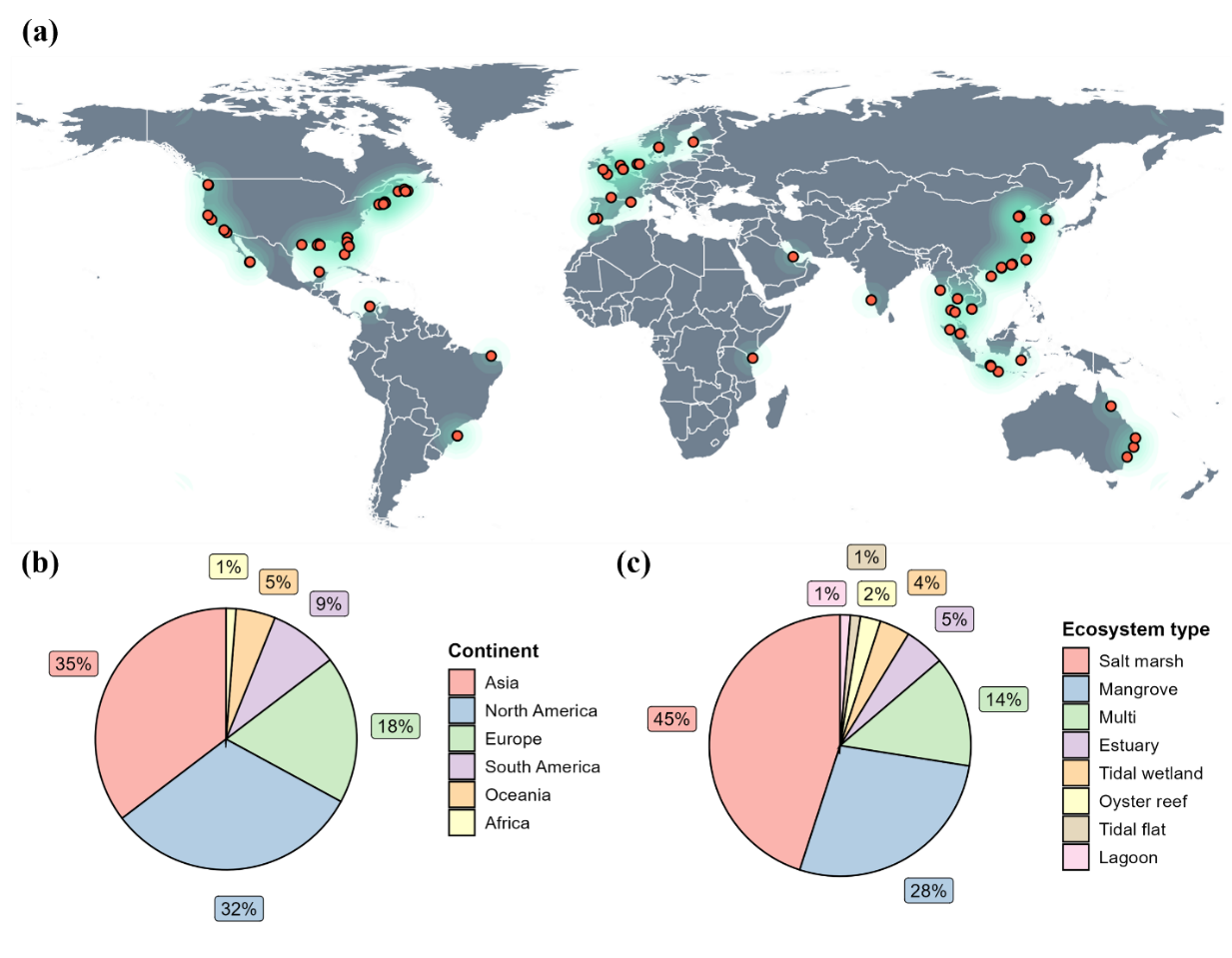


**Figure A2.** Overview of restoration clusters identified by manual extraction from a sample of 80 coastal wetland restoration articles. (a) Map of distribution and density of study sites, where each point represents a study, and the green halo indicates areas with multiple studies in proximity. (b) Study location by continent. (c) Ecosystem types addressed by restoration. “Multi” denotes a combination of salt marsh, mangrove, or estuarine ecosystem types. Map data from Natural Earth.
